# An Overview of Cell-Based Assay Platforms for the Solute Carrier Family of Transporters

**DOI:** 10.3389/fphar.2021.722889

**Published:** 2021-08-10

**Authors:** Vojtech Dvorak, Tabea Wiedmer, Alvaro Ingles-Prieto, Patrick Altermatt, Helena Batoulis, Felix Bärenz, Eckhard Bender, Daniela Digles, Franz Dürrenberger, Laura H. Heitman, Adriaan P. IJzerman, Douglas B. Kell, Stefanie Kickinger, Daniel Körzö, Philipp Leippe, Thomas Licher, Vania Manolova, Riccardo Rizzetto, Francesca Sassone, Lia Scarabottolo, Avner Schlessinger, Vanessa Schneider, Hubert J. Sijben, Anna-Lena Steck, Hanna Sundström, Sara Tremolada, Maria Wilhelm, Marina Wright Muelas, Diana Zindel, Claire M. Steppan, Giulio Superti-Furga

**Affiliations:** ^1^ CeMM Research Center for Molecular Medicine of the Austrian Academy of Sciences, Vienna, Austria; ^2^ Vifor (International), St. Gallen, Switzerland; ^3^ Drug Discovery Sciences–Lead Discovery, Bayer Pharmaceuticals, Wuppertal, Germany; ^4^ Sanofi-Aventis Deutschland GmbH, Frankfurt am Main, Germany; ^5^ Department of Pharmaceutical Sciences, University of Vienna, Vienna, Austria; ^6^ Division of Drug Discovery and Safety, LACDR, Leiden University, Leiden, Netherlands; ^7^ Department of Biochemistry and Systems Biology, Institute of Systems, Molecular and Integrative Biology, University of Liverpool, Liverpool, United Kingdom; ^8^ Novo Nordisk Foundation Centre for Biosustainability, Technical University of Denmark, Kgs. Lyngby, Denmark; ^9^ Department of Chemical Biology, Max Planck Institute for Medical Research, Heidelberg, Germany; ^10^ Axxam SpA, Bresso (Milano), Italy; ^11^ Department of Pharmacological Sciences, Icahn School of Medicine at Mount Sinai, New York, NY, United States; ^12^ Pfizer Worldwide Research, Development and Medical, Groton, MA, United States; ^13^ Center for Physiology and Pharmacology, Medical University of Vienna, Vienna, Austria

**Keywords:** solute carrier, cell-based assay, drug discovery, chemical screening, transporters, SLC

## Abstract

The solute carrier (SLC) superfamily represents the biggest family of transporters with important roles in health and disease. Despite being attractive and druggable targets, the majority of SLCs remains understudied. One major hurdle in research on SLCs is the lack of tools, such as cell-based assays to investigate their biological role and for drug discovery. Another challenge is the disperse and anecdotal information on assay strategies that are suitable for SLCs. This review provides a comprehensive overview of state-of-the-art cellular assay technologies for SLC research and discusses relevant SLC characteristics enabling the choice of an optimal assay technology. The Innovative Medicines Initiative consortium RESOLUTE intends to accelerate research on SLCs by providing the scientific community with high-quality reagents, assay technologies and data sets, and to ultimately unlock SLCs for drug discovery.

## Introduction

**FIGURE 1:**
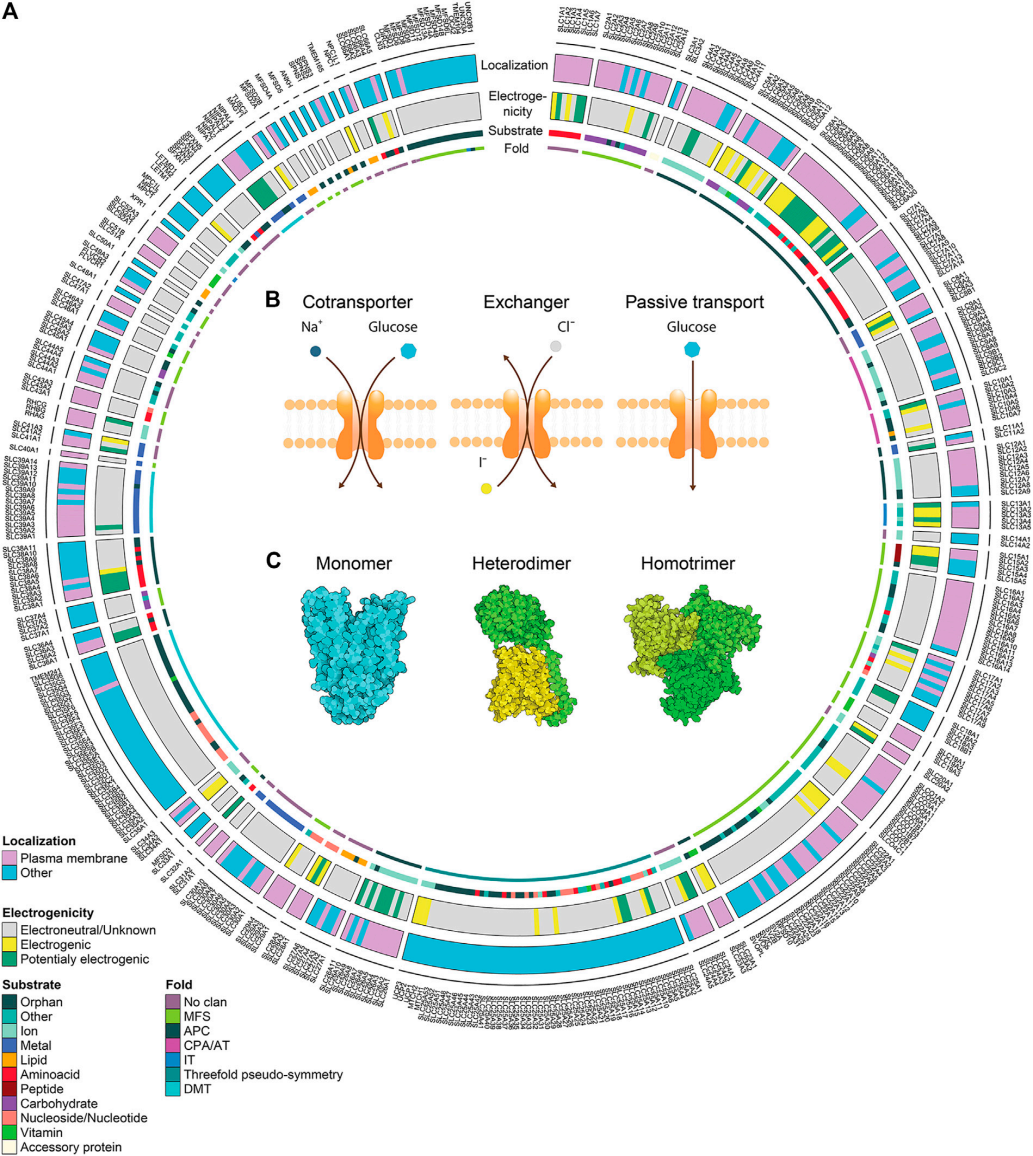
Solute carrier transporters, biochemical properties. **(A)** Schematic representation of the biochemical features of all SLC transporters. The superfamily is divided in 66 canonical sub-families and 5 non-canonical sub-families. For each SLC, the localization at the plasma membrane, the electrogenicity and the main substrate class are annotated. Annotation information regarding localization and substrate was extracted from Meixner et al. (2020) (updated by addition of SLC66 family), information regarding electrogenicity is referenced in Supplementary Table S1 and SLC fold was extracted from the Pfam database. **(B)** Transport mechanisms of SLCs. **(C)** Different association states are displayed by functional SLCs. PDB IDs 4ZW9, 6IRT and 6RVX were processed using Illustrate (Goodsell et al., 2019) to generate the visual representations.

Cells need to tightly control the chemical exchange between the intracellular and extracellular environment to maintain homeostasis, cellular integrity and safeguarding identity. Around 10% of the human genome encodes for proteins dedicated to the transport of molecules across cellular membranes, such as ATP-binding cassette transporters (ABC), ATPases, ion channels and solute carriers (SLCs) (Hediger et al., 2013). SLCs represent the second biggest group of membrane proteins and the biggest group of transporters (Höglund et al., 2011). Currently the SLC group or, better, supergroup or superfamily, as it includes proteins with different folds and phylogenetic origin, counts more than 450 members. Membership is based on either sequence or functional similarity (Figure 1A). SLCs are divided into 66 “classical” or canonical families and five new families or non-canonical families (Perland and Fredriksson, 2017; Gyimesi, 2020).

SLCs are responsible for the transport of a large spectrum of molecules including nutrients, metabolites, xenobiotics (such as phytochemicals), small molecule drugs and metal ions (Pizzagalli et al., 2020). Given the character and breadth of their substrate spectrum, it is not surprising that SLCs vary in their structure, regulation and tissue expression which is tightly coupled to the metabolic state of cells (Zhu and Thompson, 2019). This entails that SLCs are not only involved in key physiological processes, such as absorption of nutrients in the gut or ion reabsorption in kidney, but also in specialized cellular tasks, like the acidification of cytoplasm (Sedlyarov et al., 2018), amino acids sensing (Rebsamen et al., 2015; Wang et al., 2015), metal sensing (Zhang C. et al., 2020), efferocytosis (Morioka et al., 2018) or regulation of cell mass (Demian et al., 2019).

In addition, increasing amount of evidence suggests that most drugs and steroid hormones may require transporters to enter cells (Dobson and Kell, 2008; Okamoto et al., 2018; Girardi et al., 2020a). Since the expression of some SLCs is restricted only to certain tissues and cell types (O’Hagan et al., 2018), it should be possible to tailor compounds to target specific populations of cells through SLC affinity. This principle is well known from PET imaging, based on the fact that cancer cells tend to upregulate glucose transporters and glycolysis and can therefore be visualized with labelled glucose. The same principle was recently used to develop a fluorescent probe for activated macrophages (Park et al., 2019). Tailoring compounds for specific SLC-mediated drug delivery is also a promising strategy for enabling drugs to cross the blood-brain barrier (Puris et al., 2020). In addition, membrane transporters may influence the pharmacokinetic profile of a drug, and mutation or downregulation of a transporter may lead to development of resistance and treatment failure (Winter et al., 2014). Since SLCs play a role in drug-drug interactions and single nucleotide polymorphisms could affect both drug pharmacokinetics and pharmacodynamics, FDA guidelines recommend to consider these factors when evaluating drug efficiency (Giacomini et al., 2010; FDA, 2020).

At least half of the SLCs are linked to human diseases, including diabetes, gout, high blood pressure, asthma, inflammatory bowel disease, chronic kidney disease, mental disorders, cancer, and a plethora of inborn errors of metabolism, highlighting their medical relevance and therapeutic significance (Giacomini et al., 2010). In addition, recent studies reported that SLCs may be involved in the regulation of different signaling pathways involved in cancer and other diseases, such as copper transporters and MAPK pathway, or zinc transport as a modulator of Notch pathway activity (Brady et al., 2014; Nolin et al., 2019). Moreover, SLCs are acting as cellular receptors for the entry of viruses (Côté et al., 2011; Sainz et al., 2012), which can be impeded with high-affinity protein binders (Passioura et al., 2018).

Due to all the mentioned reasons and the fact that SLCs are increasingly considered amenable drug targets, the interest in SLC-oriented drug discovery is rapidly increasing (Garibsingh and Schlessinger, 2019; Avram et al., 2020, 2021; Superti-Furga et al., 2020). For instance, SLCs offer diverse structural features that favor interactions with drug-like molecules as well as appropriate accessibility to drug interactions, as more than half of SLCs are localized to the plasma membrane (Meixner et al., 2020; Pizzagalli et al., 2020). This also allows targeting SLCs with larger molecules, such as high-affinity binders, antibodies and macrocycles (Wang W. W. et al., 2019).

Despite all facts mentioned above, only a small proportion of SLCs are so far targeted by drugs or chemical probes. There are three main factors hampering the development of new chemical entities able to modulate SLC activity. First, the majority of this supergroup is relatively understudied and biological functions or substrates of many SLCs remain elusive (César-Razquin et al., 2015; Meixner et al., 2020). Second, there is a lack of high-quality biological tools, specific and reliable reagents and dedicated databases. Lastly, the number of functional assays required to study such a diverse group of targets is still limited.

To address the state of the art regarding this last point, we here provide an overview of the cell-based assay technologies currently available for SLC-focused research.

Why focus on cell-based assays and not include *in vitro* assays? For both practical and discovery strategy reasons. Practical as this overview is already sizeable as is. Strategic as we are convinced that cellular assays are better suited primary assays for proteins that are difficult to express recombinantly and purify. We consider the proper folding, natural embedding in a lipid bilayer of physiological complexity, proper cellular glycosylation pattern together with other post-translational modifications, and, most importantly, the natural repertoire and concentration of protein interaction partners, all as parameters of great importance for assessing the chemical engagement of SLC transporters. It is reasonable to assume that these parameters critically contribute to the specificity of action of individual SLCs. It is only recently that it has become possible to engineer human cells with an ease, precision and scale that has not been hitherto considered feasible (Xie and Fussenegger, 2018). Many of the assays considered in this review have been empowered by cell engineering technologies. Therefore, this review does not include assays involving recombinant, purified proteins and that are, essentially, biophysical. The review should rather serve as a guide and a starting point for choosing assay systems for anybody considering a chemical screen on SLCs or interested in studying SLC function in intact cells or at least with SLCs embedded in a cell-derived natural environment.

Assay technologies presented here are applied and developed further in the RESOLUTE consortium, a public-private partnership funded by the Innovative Medicines Initiative (IMI) of the European Union. RESOLUTE aims at empowering the research community with open-access reagents and data to unlock the SLC family for drug discovery. One of the main goals of RESOLUTE is to systematically assess the suitability of transport assay technologies for individual SLCs and develop them further (Superti-Furga et al., 2020). We are expecting to update this review with experience gained throughout the project.

### Choose Wisely: Biophysical and Biochemical Profile of Solute Carriers

SLC family members are diverse in many aspects and it is important to carefully consider features of both the SLC under study and the assay platform. This brief overview of general SLC features should act as a rationale for choosing the best-suited assay platform.

#### Transport Type

In contrast to active transporters using ATP as a source of energy, such as ABC transporters or P-Type ATPases, SLCs are transporting their substrates either in 1) facilitative mode, or 2) secondary active mode (Hediger et al., 2013) (Figure 1B). Facilitative transport is moving compounds along their own gradient, similar to ion channels. Compared to ion channels, SLCs are working in an alternating access mechanism, meaning that the SLC is actively moving its gate with a fixed stoichiometry per transport cycle, and thus SLCs have a transport rate that is several orders of magnitude smaller (Hediger et al., 2013).

Secondary active transport typically couples the movement of two different molecules. Since concentration gradients across membranes are a vital feature of cells, many SLCs take advantage of such gradients to couple the transport of different molecules. While one molecule moves along its gradient, the energy can be used to power the transport of another molecule against its gradient. Depending on the transport direction of both molecules, the SLC is either a symporter, i.e. molecules follow the same direction, or antiporter, i.e. molecules move in the opposite direction (Figure 1B). The transport rate may be proportional to the gradient of the coupled molecule. Secondary active transport is most frequently coupled to ions, mainly Na^+^, Cl^−^, K^+^ or H^+^ (Bai et al., 2018; Meixner et al., 2020), but other molecules may be coupled as well, for example SLC7A11 is exchanging glutamate for cysteine. This gives the possibility to assess changes in concentrations of coupled molecules as a surrogate of transport.

#### Solute Carrier Structural Features

Visualization of the SLCs' structure is critical for describing their transport mechanisms and molecular function. Over the past years, multiple structures of human SLCs and their homologs have been determined (Garibsingh and Schlessinger, 2019). Some SLC families have unique structures that are unrelated in evolution to structures from other SLC families (e.g. SLC1), while some SLC families are related in structure and fold (Schlessinger et al., 2010) (Figure 1A). For example, the members of the SLC7 (e.g. SLC7A5/LAT1 (Yan et al., 2019)) and SLC6 (SERT (Coleman et al., 2019)) families adopt a LeuT fold, while members of SLC2 (SLC2A1/GLUT1 (Deng et al., 2014)) and SLC16 (SLC16A7/MCT2 (Zhang B. et al., 2020)) display the MFS fold. SLCs are dynamic proteins that adopt different conformations during transport. Structural description of the transport mechanism experimentally or computationally is critical for the rational design of small molecule ligands (i.e., inhibitors, substrates, and activators). Many SLC members use an “alternating transport” mechanism, in which substrates are transported across the membrane as the protein alternates between inward-facing, occluded, and outward-facing conformations (Jardetzky, 1966). Different folds utilize different variations of this mechanisms, where commonly observed mechanisms are the “rocker switch” (e.g. SLC2), “rocking bundle” (SLC6), and “elevator” (SLC1 (Boudker and Verdon, 2010)).

#### Electrogenicity

Given the fact that many molecules transported by SLCs are charged, the transport cycle may result in charge displacement across the membrane. For example, SLC4A4 cotransports Na^+^ and HCO_3_^-^, typically in stoichiometry 1:2, and each transport cycle results in an additional intracellular negative charge. Similar observations with many other transporters open the possibility to use functional assays based on changes in membrane potential, such as electrophysiology or voltage-sensitive dyes. To the best of our knowledge, no systematic collection of the electrogenic properties of SLCs is available. We therefore collected this specific property from the literature focusing on literature for human SLCs, but also reporting data from other mammalian studies if no evidence for human SLCs was found. The literature research was based on the reviews collected in the Bioparadigm SLC tables (www.bioparadigms.org) and the original literature referenced in there. For the remaining SLCs, additional literature was collected with a special focus on SLCs that were potentially electrogenic according to their transport reaction as described in the IUPHAR/BPS Guide to Pharmacology (Armstrong et al., 2020) or the Gene Ontology (Ashburner et al., 2000; Carbon et al., 2021), or according to the description in Uniprot (Bateman et al., 2021). In total we found evidence of electrogenicity for 115 mammalian SLCs from 35 families (Figure 1A, Supplementary Table S1), corresponding to around 25% of all human SLCs. While for some SLCs the evidence of electrogenic transport – and amenability to assays based on this principle – is sufficient, for many SLCs there are no studies investigating this property, and thus the number of electrogenic SLCs is rather underestimated.

#### Redundancy

As already indicated, many SLCs are widely expressed throughout the body, while expression of other SLCs is restricted to only a few cell types (O’Hagan et al., 2018). Additionally, SLCs may have multiple isoforms, which may associate with specific cell types. These isoforms normally differ in their C- or N- termini, which may result in different protein-protein interactions (PPIs), transport efficiency, transport stoichiometry, or localization (McAlear et al., 2006; Shirakabe et al., 2006; Mazurek et al., 2010; Yoo et al., 2020).

Conversely, redundancy is also found among the substrates, as many substrates are transported by more than one SLC. For example, some 60 SLCs are thought to be competent for the transport of the 21 proteogenic amino acids (Kandasamy et al., 2018), of which approximately half is capable of shuttling glutamine (Meixner et al., 2020). As typical cell lines express around 200 different SLCs (César-Razquin et al., 2018; O’Hagan et al., 2018), more than one SLC may be potentially able to transport a particular substrate in any given cell, irrespectively of the actual subcellular localization, state of activity or actual transport rate. At the same time, while some SLCs can transport a wide range of substrates, other SLCs are specific only for one substrate. Redundancy can thus be a very challenging aspect when assaying SLCs in cellular systems. However, the cellular system can be skewed to reduce the redundancy, either by comparing several cell lines with different SLC expression profiles or by genetically alternating the levels of expression. Genetic manipulations may however introduce transcriptional and metabolic adaptations and thus potentially muddle cause and consequence when assessing individual SLCs. Such effects may be larger, the longer the cell can adapt to the genetic perturbation. Hence short-term perturbations, such as inducible systems, selective inhibitors or targeted protein degradation, may be advantageous (Bensimon et al., 2020; Wu et al., 2020). Short term perturbations may be also used as a control to set up the assay since the availability of selective inhibitors for SLCs is limited. Alternatively, the wild-type SLC can be compared with the SLC bearing a transport-deficient mutation.

#### Localization

Cellular localization of a particular SLC is a crucial consideration for assay choice. Some SLCs transport molecules only across the membranes of intracellular compartments, like the SLC25 family expressed on the mitochondrial membranes, while others are not restricted to only one organelle, such as SLCs with multiple isoforms, which are expressed in different organelles (Mazurek et al., 2010; Yoo et al., 2020). Annotation of SLC localization based on literature search revealed that around half of the SLCs are localized at least partially to the plasma membrane (Meixner et al., 2020) (Figure 1A). Since many assay technologies measure changes in substrates at the whole-cell level, special attention should be devoted to the choice of an assay for an intracellularly localized SLC. This limitation can be overcome by artificially redirecting intracellular SLCs to the plasma membrane (Lisinski et al., 2001; Forbes and Gros, 2003; Wang Y. et al., 2019). However, redirection will also alter parameters, such as local ion gradients. Thus, assays that are compatible with the intracellular localization, such as for example assays based on genetically encoded sensors, or fluorescent substrates, are generally preferable. Alternatively, some of the intracellular SLCs can be assayed in permeabilized cells (Kuznetsov et al., 2008), or organelles isolated using techniques such as LysoIP (Abu-Remaileh et al., 2017) or MitoIP (Chen et al., 2017). This approach was recently used to characterize SLC localized in melanosomes (Adelmann et al., 2020).

#### Regulators/Modulators of Solute Carrier Function and Localization

To function properly, many SLCs require chaperones, oligomerization or interaction with other proteins, which may regulate their function in several ways. Protein-protein interactions (PPIs) play an important role in subcellular localization. While localization of some SLCs is determined by a signal peptide, other SLCs require more extensive interactions for trafficking. For instance, members of SLC16 family require chaperone proteins basigin (CD147) or embigin (gp70) for translocation to the plasma membrane (Felmlee et al., 2020). Other SLCs may be restricted to vesicles, and only after a secondary signal will be translocated to the plasma membrane, such as the insulin responsive glucose transporter SLC2A4 (Jaldin-Fincati et al., 2017).

Some SLCs necessitate oligomerization for functioning (Figure 1C). Heteromerization is required, for example, for SLC families 3 and 7 (Fotiadis et al., 2013), 51 (Ballatori et al., 2013) and 54 (Herzig et al., 2012). Other SLCs form homomers, such as SLC4A4 forming homodimers (Huynh et al., 2018), or SLC1A3 forming homotrimers (Canul-Tec et al., 2017). However, the importance of homomerization for transporter function may vary. A recent study on SLC2A1 employing super-resolution microscopy suggested that SLCs may form dynamic clusters of different size with distinct transport activity (Yan et al., 2018). A similar phenomenon was observed with SLC16A7, where homodimerization increased transport activity, suggesting cooperativity between two subunits (Zhang B. et al., 2020).

PPIs are also important for modulation of SLC function. Known positive regulators include IRBIT, a regulator of SLC9A3, SLC4A4 and SLC26A6 function (Ando et al., 2014), or MAP17 regulating SLC5A2 (Coady et al., 2017). Among known negative regulators are PASCIN1 for SLC12A5 function and expression (Mahadevan et al., 2017), the ubiquitin ligase RNF5 for SLC1A5 and SLC38A2 (Jeon et al., 2015), and OS9 for ER-associated degradation of SLC12A1 (Seaayfan et al., 2016). As these interactions typically do not happen in isolation, but in the complex cellular environment where many SLCs exist in large multi-protein complexes, different interactors may affect transport functions in different ways (Haase et al., 2017; Mahadevan et al., 2017) and they may influence assay settings or production of recombinant protein for *in vitro* assays (Kost et al., 2005). At the same time, interactions can be explored for indirect pharmacological modulation of SLC function.

Post-translational modifications (PTMs), such as glycosylation, SUMOylation, phosphorylation or acetylation, regulate function and trafficking of certain SLCs (Pedersen et al., 2016; Czuba et al., 2018). Importantly, glycosylation can affect drug binding (Hoover et al., 2003). Other factors that may modulate the transport function are for example pH (Webb et al., 2016), membrane potential or binding of small molecules to intracellular non-substrate binding sites (Scalise et al., 2015; Windler et al., 2018). Additionally, SLC mediated transport can be slowed down by decreasing the temperature, which can be exploited in assay development.

### Select Carefully: Assay Throughput and Chemical Space

A key factor for a novel drug discovery campaign is the selection of compounds for screening, which determines the throughput capacity required from an assay. While large chemical libraries can be successfully screened only in high throughput (HTP) assays, capable of testing millions of compounds, focused chemical libraries can be screened effectively with lower throughput (LTP). HTP assays typically implement simple protocols and their quality is primarily determined based on the Z’ factor, which quantifies the assay window (Zhang et al., 1999). Importantly, HTP assays require often special instrumentation, rigorous assay optimization and follow-up secondary screening campaigns to validate the results (Walters and Namchuk, 2003). Assays with LTP may require less optimization and sometimes also provide more information (e.g. kinetics). LTP assays can be sufficient as a secondary screening assay in chemical screening campaigns, or if a transporter for a selected substrate or a drug is investigated (Yee et al., 2019). Importantly, many LTP assays can be adapted to HTP mode. An interesting compromise between library size and the chemical space bias are fragment-based approaches, shown to be applicable to SLCs (Parker et al., 2017a). Alternatively, to reduce the number of compounds for experimental validation, large compound libraries can be pre-screened using virtual screening approaches.

Virtual screening, a computational approach, is an efficient approach to evaluate the activity of large compound libraries against a specific protein. Virtual screening can be grouped into ligand-based approaches where an algorithm is developed based on a known set of small molecule ligands, and structure-based virtual screening or molecular docking that evaluates the complementarity between small molecules and an experimentally determined SLC structure or a computational model. Ligand-based approaches have been used to identify small molecules for a range of SLC targets (reviewed in (Türková and Zdrazil, 2019)). One limitation of ligand-based approaches is the availability of known active compounds to develop predictive models.

Alternatively, molecular docking on a 3D molecular structure is commonly used to predict activity of relatively unbiased, and often massive, compound libraries, which is critical for identifying novel chemical scaffolds (Irwin and Shoichet, 2016). A combination of virtual screening and focused chemical libraries employing LTP assays might be a powerful approach to reveal promising drug candidates (Geier et al., 2013; Huard et al., 2015). However, this approach is limited to SLCs with sufficient structural information to warrant meaningful docking models. Recently it was shown that combining both ligand- and structure-based approaches can be a powerful approach to identify SLC drug interactions (Schlessinger et al., 2018) such as the case of SLC22A24 deorphanization (Yee et al., 2019).

Importantly, the choice of the chemical library, as well as screening technology depends on the availability of resources, including budget, platforms, instruments, and chemistry.

## Cell-Based Assay Technologies for Solute Carrier Oriented Screening

The choice of the most suitable assay is not only dictated by the characteristics of a particular SLC, but also by the goal of the screening. While many biological questions related to the role of individual transporters in biological processes can be answered only in animal models (Jiménez-Valerio et al., 2016; Pisarsky et al., 2016; Nakata et al., 2020), or with different approaches, such as genetic screens (Fauster et al., 2018; Kory et al., 2018; Sedlyarov et al., 2018; Girardi et al., 2020a), this review will focus on target-based cellular technologies for chemical screening in drug discovery campaigns or mechanistic transport studies. Most presented assays are best suited for *in vitro* applications, which may limit physiological relevance. However many assays can be applied, for example, with *ex vivo* isolated tissues or perfused organs. Since SLC function is determined also by concentration gradients, the assay system can be brought closer to physiological conditions by for example using physiological medium (Cantor, 2019; Rossiter et al., 2021).

In the next section, we outline key considerations and provide an overview of a range of assay technologies that we have successfully adopted for SLCs. Without going into experimental details, we summarize information on the principles operating within the assays, some parameters to consider and some SLC families for which the assay may be particularly suitable. The reader is referred to the literature for further information.

**FIGURE 2:**
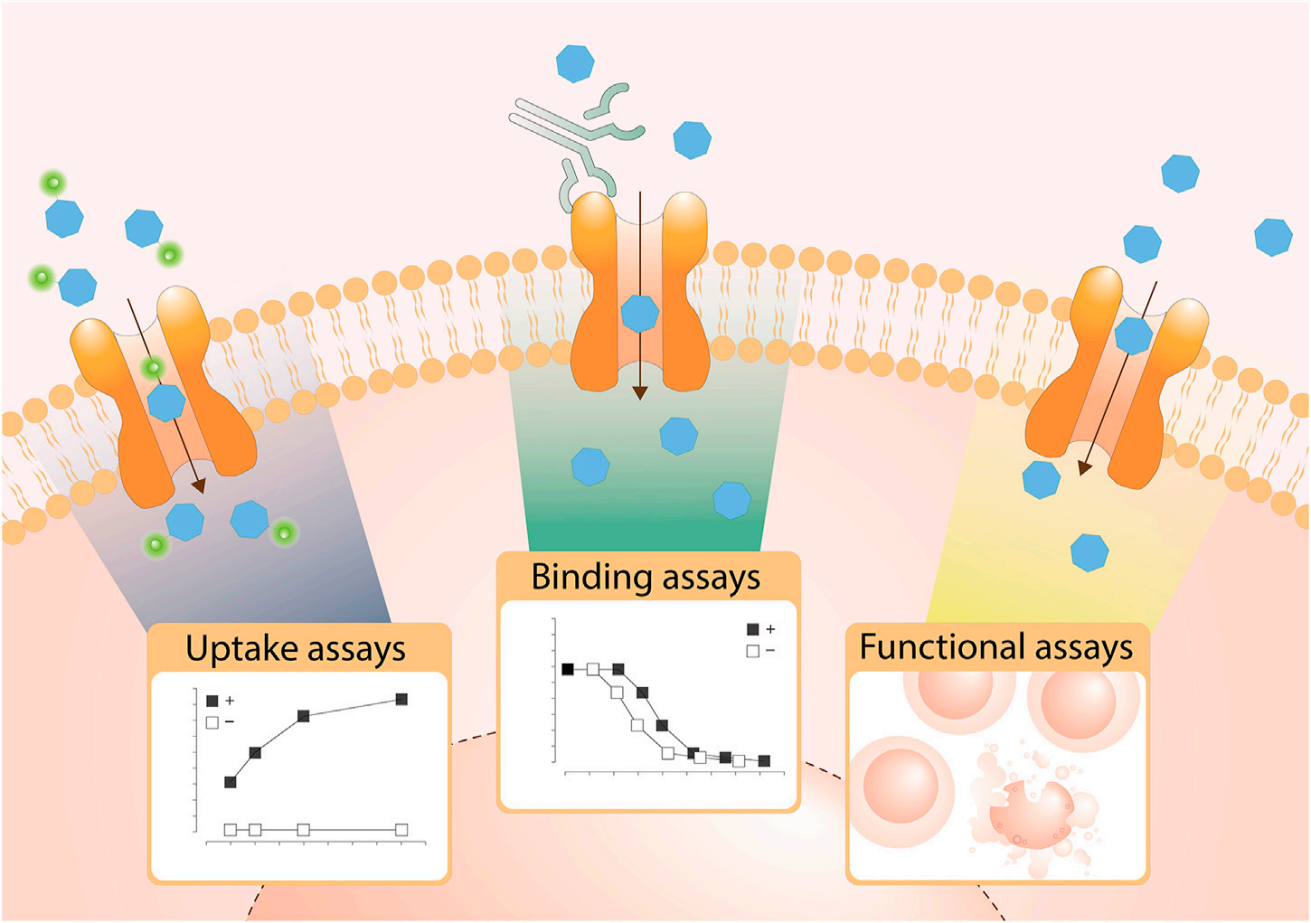
Overview of the types of cell-based transport assays described in this review. Uptake assays directly measure the changes in the transported substrate across a cellular membrane. Binding assays report on protein stabilization upon binding of a molecule to the SLC in a cellular environment. Functional assays assess secondary effects in cells as a consequence of substrate transport.

Assays are divided based on their assay principle (Figure 2, Table 1) (Wang W. W. et al., 2019). Cell-based substrate transport assays are more suitable to screen for SLC inhibitors or to connect the SLC to its substrate; while binding assays can identify molecules that bind to the SLC but not necessarily alter transport. These could be further developed as chemical modulators of function, as corrector, potentiator, stabilizer or degrader depending on the target SLC (Gerry and Schreiber, 2020; Lopes-Pacheco, 2020). Functional assays can uncover SLC inhibitors, as well as modulators of transporter function, and thus can be advantageous when screening for SLC activators.

**TABLE 1:** Overview of assays presented in this review. Examples of intracellular SLCs are highlighted in bold.

	Assay	Special technical requirements	SLC suitability	Level of throughput	Advantages	Limitations
Substrate uptake assays	Radioligand uptake assay	Radiolabeled SLC substrate	Widely suitable (e.g. SLC1, SLC2, SLC6, SLC7, SLC10, SLCO, SLC13, SLC22)	Low	1) Versatility	1) Radioactive readout 2) Cannot distinguish inhibitors from substrates
	Fluorescent substrate uptake assay	Fluorescent SLC substrate	Widely suitable (e.g. SLC6, SLC10, SLC18, SLC27, SLCO, SLC22, SLC47, **intracellular SLCs with microscopy readout**)	High	1) Simple setup 2) Kinetics	1) Not suitable for testing of compounds with fluorescent or quenching properties
		Genetically encoded biosensors	GE biosensor	Widely suitable (e.g. SLC1, SLC2, SLC5, SLC26, SLC12, SLC16, SLC42, **SLC54**)	Medium to high (sensor and readout dependent)	1) Possibility to target the sensor to a specific subcellular compartments 2) Dynamic range and sensitivity 3) No need of cell loading with dyes 4) Temporal resolution	1) Robust expression of the sensor is required
			MS-based transport assays for metabolites or ion trace elements	Mass spectrometer (ICP-MS for ion trace elements)	Applicable to most SLC families **(intracellular SLCs upon organelle isolation)**	Low	1) Detection of multiple analytes 2) Specificity and direct measurement of substrates	1) Specialist knowledge required
Binding assays	Thermal shift assay		Widely suitable (e.g. SLC2, SLC16)	Low to medium	1) Direct protein-ligand interaction 2) Label free 3) Versatility	1) Not all ligands will shift Tm 2) Possible loss of interaction due to high T 3) Prone to false negative results
Functional assays	Fluorescent dyes	FLIPR/Hamamatsu FDSS (or similar) plate reader	Widely suitable (e.g. SLC1, SLC4, SLC6, SLC9, SLC12 SLC16)	High	1) Simple protocols 2) Flexibility 3) Good dynamic range 4) Temporal resolution	1) Loading of cells with dyes 2) High costs
	Electrophysiology	Patch clamp experimental setup	Electrogenic SLCs in plasma membrane (e.g. SLC8)	Low	1) High accuracy 2) Real-time measurement 3) Single-cell analysis	1) Limited to electrogenic SLCs 2) Small signal window
	SSM-based electrophysiology	SURFE2R	Electrogenic SLCs (e.g. SLC1, SLC8, SLC15, SLCO, **intracellular SLCs upon organellar membrane isolation**)	Low to medium	1) High accuracy 2) Real-time measurement 3) High signal amplification	1) Membrane potential cannot be applied 2) Limited usability if transporter function depends on PPI
	SLC-GPCR coupling		Limited to SLCs transporting GPCR ligands (e.g. SLC63, SLC59)	High	1) Specificity and sensitivity	1) Many steps requiring optimization and posing confounding factors 2) Risk of false positive/negative hits
		Label-free impedance-based assay	xCELLigence real-time cell analyser	Limited to SLCs transporting GPCR ligands (e.g. SLC6, SLC29)	High	1) Label-free and non-invasive 2) Real-time measurement	1) Prone to false positive/negative hits
			SLC coupling to nuclear hormone receptor		Limited to SLC transporting nuclear hormone ligands (e.g. SLC10, SLC16, SLCO, SLC22)	High	1) Unmodified SLC substrate 2) Real-time measurement	1) Redundant SLC expression may limit usability
			Phenotypic assay		Widely suitable (e.g. SLC16, **SLC25**)	High	1) Viability readout 2) High specificity in case of reciprocal interaction	1) Prior knowledge of a strong genotype-phenotype connection required

### Substrate Uptake Assays

The most commonly used strategy to assess SLC transport function are substrate uptake assays (Wang W. W. et al., 2019). This approach directly assesses the transport function by measuring the changing concentrations of a transported molecule extra- and intracellularly (Figure 2). Cellular systems are most widely used, but uptake assays can also be performed in vesicles, such as liposomes or in microinjected oocytes from *Xenopus laevis* (Nimigean, 2006).

#### Radioligand Uptake Assay

Radioligand uptake assays are vastly employed to study the structure and function of transporters. In general, a radiolabeled substrate is used to quantitatively study the substrate uptake across the plasma membrane into a closed compartment (e.g. whole cells, perfused organs, tissue pieces, synaptosomes, vesicles) (Sucic and Bönisch, 2016). The inhibitory potency of a ligand is probed through the competition with the radiolabeled substrate. The transporter of interest can either be endogenously expressed in a native system or heterogeneously expressed. Transient expression in diverse cell lines has become increasingly popular because different cloned transporters can be probed under the same assay conditions. Additionally, site-directed mutagenesis studies can be performed. This makes radioligand uptake assays an excellent tool to study the molecular determinants governing activity and selectivity of a compound. It is noteworthy that radioligand uptake assays measure a functional effect and the obtained activity values, typically IC_50_ values, do not directly reflect the affinity of the tested compounds.

##### Technical Requirements and Level of Throughput

A radiolabeled substrate, typically ^3^H labeled, is required to perform the assays. Such radioligands are either commercially available (e.g. for the monoamine transporters SLC6A2-4 transporters ^3^H-norepinephrine, ^3^H-dopamine, ^3^H-imipramine, respectively) (Sucic and Bönisch, 2016) or they can be synthesized as demonstrated for the GABA transporter SLC6A12 and the creatine transporter SLC6A8 (Al-Khawaja et al., 2018). In order to measure the amount of radioactive substrate, which was transported inside the cells, the cells are lysed, a scintillation cocktail is added, and the plates are analyzed with a scintillation counter. Performing radioligand uptake assays requires multiple washing steps which results in LTP.

##### Experimental Setup

Sucic and Bönisch have described in detail how to perform radioligand uptake assays with special focus on neurotransmitter transporters (Sucic and Bönisch, 2016). On the day prior to the uptake experiment, the cells are plated into multiple well plates, which were precoated with Poly-D-Lysine, to ensure attachment of the cells to the plate. On the day of the uptake assay, the wells are washed multiple times with buffer and are then incubated with the radiolabeled substrate together with different concentrations of inhibitors. Additionally, positive and negative controls are performed by incubating wells with a high concentration of the radioligand to measure maximum inhibition as well as with buffer to measure nonspecific inhibition. The uptake is stopped by multiple washing steps with ice-cold buffer. Finally, the cells are lysed, a scintillation cocktail is added, and the plate is analyzed with a scintillation counter. Data analysis is typically performed by fitting the data to a sigmoidal-dose response model by applying nonlinear-regression in order to obtain IC_50_ values. K_i_ values can be calculated for competitive inhibitors according to the Cheng-Prusoff equation (Cheng and Prusoff, 1973).

The amount of radiolabeled substrate, the number of plated cells and the incubation time highly depend on the nature of the transporter and need to be optimized accordingly.

##### Suitable Solute Carrier Families

Radioligand uptake assays have been widely employed to study diverse SLC families, including: SLC1 (Garibsingh et al., 2018), SLC2 (Tripp et al., 2017), SLC6 (Borden, 1996; Núñez et al., 2000; Al-Khawaja et al., 2014, 2018; Hofmaier et al., 2014; Richter et al., 2019), SLC7 (Chien et al., 2018), SLC10 (De Bruyn et al., 2011), SLCO (De Bruyn et al., 2011), SLC13 (Colas et al., 2017), or SLC22 (Erdman et al., 2006).

##### Assay Advantages, Limitations and Approximate Costs

The advantage of radioligand uptake assays is that different transporters as well as mutants can be measured in the same assay set-up under the same conditions. A disadvantage of the assay is that only a functional effect is measured and not the actual binding affinity. For measuring binding affinities other assays such as Surface Plasmon Resonance (SPR), isothermal titration (ITC) (Rajarathnam and Rösgen, 2014) or radioligand binding assays (Sucic and Bönisch, 2016) can be utilized. Another profound shortcoming of the radioligand uptake assay is that it cannot distinguish between inhibitors and substrates. The actual costs for performing the assay depend highly on the cost of the radioligand.

#### Fluorescent Substrate Uptake Assay

Fluorescent surrogate substrate assays rely on transport of a fluorescently labeled analogue of an SLC’s natural substrate (e.g. a BODIPY-labeled fatty acid, an Alexa-labeled peptide) or a fluorescent drug or dye which acts as an alternative substrate of the SLC (Fardel et al., 2015; O’Hagan and Kell, 2020). This approach allows to monitor the activity of SLCs in cells in real time.

##### Technical Requirements and Level of Throughput

This assay strategy requires a fluorescently labeled substrate and a conventional fluorometric microplate reader, ideally allowing for real time monitoring of fluorescence changes within the cell. For HTP screening, a reader compatible with 384 or 1,536 well plates and with integrated robotic handling systems is advantageous. Alternatively, cells can be analyzed by microscopy on single slides or in multi titer plates by high content imaging to visualize the distribution of the fluorescent substrate within intracellular compartments.

##### Experimental Setup

A cell line overexpressing the SLC of interest in an inducible or constitutive manner is generated for comparing SLC-mediated and unspecific substrate uptake in the same cellular background. To run the assay, the growth medium is removed, and cells are incubated with transport buffer. In case of sym- or antiporters, this buffer should contain relevant ions which are co-transported along with the fluorescent substrate. In addition, a cell-impermeable quenching agent can be added to the buffer to eliminate extracellular fluorescence and thus enhance the signal-to-noise ratio (Wemhöner et al., 2006; Zhou et al., 2010). Finally, the fluorescent substrate is added, and its uptake is monitored for several minutes. In presence of a quencher, the assay can be run in a homogenous format and uptake can be monitored continuously. Without addition of a quencher, washing steps with transport buffer need to be included after substrate addition to remove the remaining fluorescent substrate from the extracellular space.

For assay optimization, cell clones are selected based on the signal-to-background ratio of fluorescent substrate uptake and – if available – the observed activity of known potent and selective tool compounds. Further clone selection criteria include qPCR and western blotting to quantitate protein expression levels. To find optimal assay conditions, the K_m_ of the fluorescent surrogate substrate is estimated (Wittwer et al., 2013), and competition experiments with the unlabeled physiological substrate can be performed.

##### Suitable Solute Carrier Families

Fluorescent surrogate substrate uptake assays are used broadly and have successfully been applied to various SLC families such as SLC6 (Zwartsen et al., 2017), SLC10 (Mita et al., 2006), SLC18 (Hu et al., 2013), SLC27 (Sandoval et al., 2010; Zhou et al., 2010), SLCO and SLC22 (Fardel et al., 2015), and SLC47 (Yasujima et al., 2010; Fardel et al., 2015)

##### Assay Advantages, Limitations and Approximate Costs

Fluorescent surrogate substrates offer the advantage of performing a rapid, simple and homogenous assay without washing steps, if performed in presence of a quenching agent. Thus, fluorescent surrogate substrate assays are amenable for HTP screening aiming for rapid characterization of lead compounds and can replace more laborious and cost intensive approaches like using radiolabeled substrates or isolation and fractionation of natural substrates. Furthermore, the activity of SLCs can be monitored in real time and cellular process such as trafficking, sequestration or compartmentalization of fluorescent solutes can be visualized. The limitations of this strategy include the need to identify a fluorescent surrogate substrate, which is likely not feasible for every SLC. Also, compounds which are autofluorescent or fluorescence quenchers can interfere with the readout. The costs of the assay largely depend on the costs of the surrogate substrate.

#### Genetically Encoded Biosensors

**FIGURE 3:**
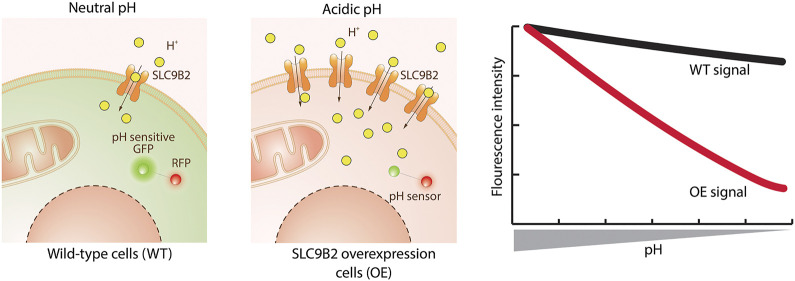
Transport assay using a genetically encoded biosensor. The exemplified assay uses a protein sensor to detect changes in cellular pH caused by the substrate transported by the SLC. The sensor encodes a pH sensitive green fluorescent protein (GFP) linked to a red fluorescent protein (RFP – used for normalization). Wild-type (WT) cells have a neutral cytoplasmic pH where GFP is active. Upon overexpression of SLC9B2 (a proton importer) and addition of its substrate, the increased concentration of protons lowers the cytoplasmic pH. This causes the quenching of the GFP and therefore a decrease in fluorescence intensity compared to WT cells.

Genetically encoded fluorescent biosensors are proteins that bind an analyte or sense a physical property and translate its concentration into a change in fluorescence, either intensiometric or ratiometric. Beyond the well-known calcium- and voltage-indicators such as the GCaMP (Dana et al., 2019) and ASAP (Villette et al., 2019) series, biosensors for a diverse array of cellular analytes now exist (Greenwald et al., 2018). Since biosensors can be specifically targeted to cellular compartments by appropriate targeting motifs, they hold promise to measure intracellular transport. Biosensors measuring ions such as Ca^2+^, Cl^−^ or H^+^, as for example GCaMP (Nakai et al., 2001), SuperClomeleon (Kuner and Augustine, 2000; Zhong et al., 2014) and pHluorins (Miesenböck et al., 1998), are especially suitable for the assessment of SLC transport, either by detection of the primary substrate or coupled ions (Figure 3).

##### Technical Requirements and Level of Throughput

A cell line co-expressing the SLC of interest together with a biosensor for the transported substrate is required. The change in fluorescence can be detected by microscopy, flow cytometry or using a plate reader. Ideally, instruments are equipped with perfusion or injection modules to enable a time-resolved study of transport. Plate-based measurements of biosensors are generally applicable to HTP screening by the use of plate readers such as FLIPR or Hamamatsu FDSS that can accommodate 384-well plates format.

##### Experimental Setup

For a successful assay, the cell line should be optimized for homogenous and stable expression levels of both SLC and biosensor, as both will influence the dynamic range. The gene coding for the biosensor is always introduced exogenously and its expression should be examined for correct subcellular targeting and absence of overexpression or folding artifacts. Before the experiment, cells can be starved or treated with drugs to deplete intracellular levels of transporter substrate. Next, cells are incubated in an appropriate assay buffer containing test compounds (e.g. drug candidates). In the case of intensiometric biosensors, a first measurement needs to be performed for normalization. Then, the substrate is added, and the resulting fluorescence change is either recorded immediately to measure kinetics of the transport reaction or with a time-delay to measure the steady-state level. Fitting the concentration of the externally supplied substrate against the fluorescence change results in an apparent K_0.5_, or IC_50_, representing the combination of biosensor affinity, transporter properties and metabolic conversion of the substrate. Alternatively, the substrate concentration can be held constant while varying the test compound concentration for IC_50_ determinations. As an additional benefit of ratiometric biosensors, the fluorescence change can be converted into absolute intracellular concentrations with the requirements of careful calibration and ratio-processing (Hou et al., 2011; Pomorski et al., 2013).

##### Suitable Solute Carrier Families

Biosensors can be widely applied to study SLCs which transport ions or metabolites detectable by a biosensor (Sanford and Palmer, 2017). Biosensors were successfully employed in a number of assays for glucose transporters from families SLC2 and SLC5 (Takanaga and Frommer, 2010; Keller et al., 2019), chloride transport mediated by the SLC26 family (Galietta et al., 2001; Zhong et al., 2014), iodide transported by the SLC12 family (Valdez-Flores et al., 2016), glutamine transported by the SLC1 family (Gruenwald et al., 2012), pyruvate transported by the SLC54 family (Arce-Molina et al., 2020), pyruvate and lactate transported by the SLC16 family (Contreras-Baeza et al., 2019) and ammonium ions transported by yeast homologues of SLC42 family (Ast et al., 2017).

##### Assay Advantages, Limitations and Approximate Costs

Biosensors can directly measure the concentration of the substrate, offer temporal resolution and can be targeted to cellular compartments. Biosensors overcome the need of cell loading with chemical organic dyes, potentially affecting cell physiology. Biosensor-based assays are inexpensive and essentially have the costs of running a cell culture. A practical disadvantage can be the requirement of the expression of two genes, transporter and biosensor. The main limitation is the availability of biosensors, most of which have been developed to study signaling events and not for transport measurements. However, these can be optimized or repurposed for transporter assays. For instance, a popular class of biosensors is expressed on the plasma membrane for measuring release of neurotransmitters, such as glutamate (Marvin et al., 2013), GABA (Marvin et al., 2019), glycine (Zhang et al., 2018), dopamine (Patriarchi et al., 2018; Sun et al., 2018), norepinephrine (Feng et al., 2019), acetylcholine (Jing et al., 2018), and serotonin (Wan et al., 2020). These biosensors could be co-expressed with SLCs transporting their ligand, similar to coupling of SLC transport to G protein coupled receptor (GPCR) downstream signaling (Vlachodimou et al., 2019). After ligand application and SLC-dependent transport into cells, the reduction of extracellular ligand concentration will decrease the biosensor’s apparent affinity. In fact, pharmacological inhibition of SLCs involved in the clearance of glutamate were measurable with biosensors and support this assay strategy (Armbruster et al., 2016, 2019; Pinky et al., 2018; Wan et al., 2020).

#### Mass Spectrometry-Based Transport Assay

**FIGURE 4:**
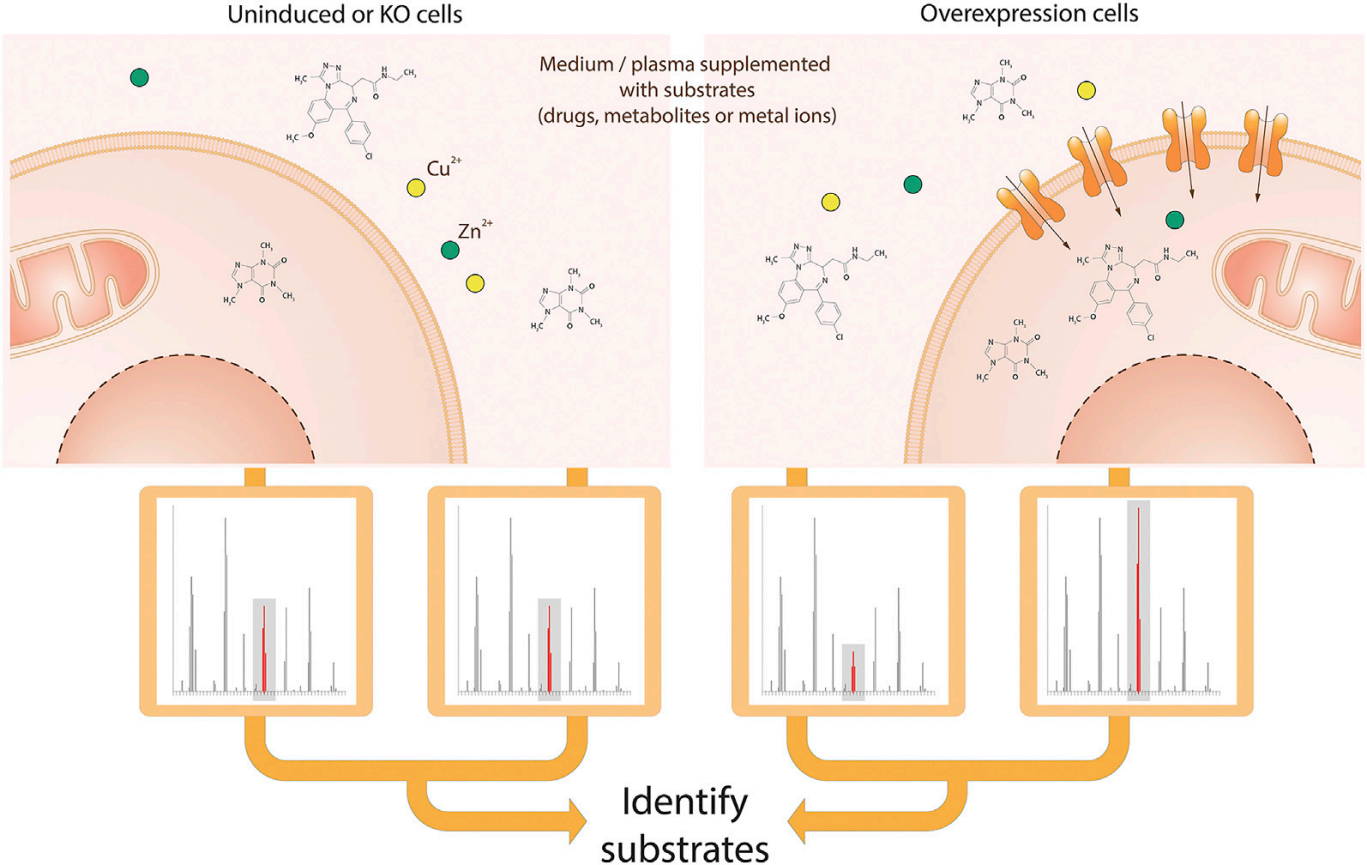
Schematic view of the MS-based transport assay for SLCs. Cells are incubated in medium or plasma containing a mix of metabolites, drugs and ions. After incubation, medium and/or intracellular fractions are extracted and prepared for MS analysis, followed by alignment and identification of molecules or ions. Both the comparison of identified molecules or ions in cellular extracts and medium as well as the comparison of cells with the SLC of interest knocked-out and overexpressed enable the identification of the metabolites, drugs or ions that are transported by the SLC of interest.

Mass spectrometry (MS) is an analytical technique that measures the mass to charge ratio (m/z) of molecules present in samples. These measurements are used to calculate the exact molecular weight of components and thus identify and quantify the compounds in the sample. This technique is widely used in metabolomics, the study of low molecular weight molecules that take part in metabolic reactions required for maintenance, growth and function of cells (Oliver et al., 1998). Metabolomics analysis by MS is a powerful tool to determine transporter substrates by measuring the uptake or excretion of small molecule compounds by cells (Figure 4).

##### Technical Requirements and Level of Throughput

The assay requires incubation of cells or organelles in an appropriate medium for a short period of time. Comparison of the uptake of compounds by genetically engineered cell lines with SLC knock-out or overexpression (Gründemann et al., 2005), as well as the manipulation of uptake medium conditions (e.g. pH, addition of inhibitors or other compounds for competition (Dickens et al., 2018)) can facilitate the identification of transporter substrates. Subsequently samples must be appropriately prepared for mass spectrometric analysis (Dunn et al., 2011; Vuckovic, 2012).

Cajka and Fiehn provide an excellent review of the various MS technologies available for metabolomics, along with advantages and limitations (Cajka and Fiehn, 2016). Untargeted MS following methodologies and guidelines in (Brown et al., 2005; Broadhurst and Kell, 2007; Dunn et al., 2011; Mullard et al., 2015; Broadhurst et al., 2018) enable the measurement of differences in the uptake of a broad range and number (thousands) of compounds by cell lines (Wright Muelas et al., 2020). Targeted MS can alternatively be used, limiting the number of compounds measured (typically <200) but at the same time enabling absolute quantification. Throughput depends on the approach and instrumentation used.

##### Experimental Setup

The following steps describe the preparation of intra- and extracellular samples for MS analysis over a time course (Wright Muelas et al., 2020). Following incubation of cells in uptake medium, spent medium is collected after centrifugation, followed by extraction using methanol. The remaining cell pellet is washed, followed by quenching and extraction of intracellular metabolites using methanol. The spent medium and intracellular extracts are subsequently lyophilised, and reconstituted in water ready for analysis by LC-HRMS/MS.

##### Suitable Solute Carrier Families

Mass spectrometry analysis of transporter substrates is applicable to most SLC families.

##### Assay Advantages, Limitations and Approximate Costs

Advantages of the assay are that sampling over a period of time enables transport kinetics to be measured. Untargeted metabolomics allows measurement of relative changes in a wide range of compounds, known and unknown, potentially leading to novel substrate identification. Whilst fewer compounds can be reliably measured using targeted techniques, these enable quantification of the changes in specific compounds to be measured. A disadvantage with both methods is the requirement for expensive instrumentation with specialist knowledge and skills required to run and maintain, along with complex data processing and analysis. However, these disadvantages are outweighed by the wealth of information provided by these assays. This experimental set up is particularly well suited to SLCs expressed at the plasma membrane. Similar approaches have been reported for SLCs localized in cellular organelles such as lysosomes and mitochondria, but require additional cell line engineering to enable the pulldown approach ensuring a quick and efficient organelle isolation (Abu-Remaileh et al., 2017; Chen et al., 2017).

#### Mass Spectrometry-Based Analysis of Intracellular Ionic Trace Elements

Trace elements in their ionic form mediate biochemical reactions in human cells by acting as enzyme cofactors or centers for stabilizing protein structures. Deficit or accumulation of these substances lead to cell toxicity and severe diseases in humans and therefore, intracellular trace ion concentrations (i.e. the “ionome”) must be tightly controlled. Inductively coupled plasma mass spectrometry (ICP-MS) is the most sensitive method able to determine and quantify the human “ionome” by detecting isotopes at a very low concentration. The analytical technique is widely used in the pharmaceutical industry to detect and quantify elemental impurities. However, in recent studies, ICP-MS also enables to profile trace elements in mammalian cells (Malinouski et al., 2014; Tsai et al., 2014; Konz et al., 2017). Using cell lines with a genetically deleted or artificially overexpressed SLC allows a systematic identification of SLCs involved in the transport of ions (Figure 4).

##### Technical Requirements and Level of Throughput

Ionomics assays use mammalian cells overexpressing or bearing knock-out genes encoding particular transporters to quantify the change of inorganic ions by ICP-MS upon cell lysis. ICP-MS-based ionomics is rather a LTP assay, as a significant volume of sample is required.

##### Experimental Setup

To analyze the amount of intracellular ions present in the sample (i.e. ^23^Na, ^24^Mg, ^31^P, ^32^S, ^39^K, ^44^Ca, ^51^V, ^52^Cr, ^55^Mn, ^56^Fe, ^59^Co, ^60^Ni, ^63^Cu, ^66^Zn, ^97^Mo), HEK293 cells stably expressing SLC transporters under the control of a doxycycline inducible promoter are grown in standard cell culture medium, naturally containing a selection of ions and metals. Transporter expression is induced by overnight addition of doxycycline. The next day cells are thoroughly washed with an isotonic Tris/choline-chloride based wash buffer, to completely remove all extracellular ions and subsequently lysed with a Tris/choline-chloride/Triton X-100 based lysis buffer, not containing any of the measured ions. The sample is then ionized by the inductively coupled plasma and the ions are transferred to the mass spectrometer, where they are separated based on their mass-to-charge ratio (m/z). The detector receives a signal proportional to the quantity of ions present in the sample. The ion intensities are normalized to either cell lysate protein concentration or intensity of ^31^P, which were shown to change linearly with the number of cells harvested. To evaluate the contribution of a particular SLCs in the transport of inorganic ions, the normalized intensity ratios of the ions are compared between HEK293 cells with or without induced overexpression of a particular SLC.

Due to the sensitivity of ICP-MS, an exhaustive optimization of washing steps, cell lysis, cell count normalization, ion detection and statistical analysis is required to precisely detect intracellular ion levels (Malinouski et al., 2014; Konz et al., 2017).

##### Suitable Solute Carrier Families

This assay format was shown to be suitable for both efflux and influx transporters of metal ions and metalloids including, but not limited to, the aforementioned ions (Malinouski et al., 2014; Konz et al., 2017). Examples of these transporters are from families SLC11, SLC30, SLC31, SLC39, and SLC40. Furthermore, we speculate that SLCs for which the main substrate transport is driven by metal ions detectable with ICP-MS may also be amenable to this assay technology.

##### Assay Advantages, Limitations and Approximate Costs

Determination of the intensities of the monoisotopic ion ensures specificity and allows direct measurement of inorganic SLC substrates. The method also allows normalization based on either protein concentration of cell lysates or amount of ^31^P with the limitation in case of studies with phosphate transporters. Furthermore, the assay was demonstrated to be suited for SLCs located on the plasma membrane or endoplasmic reticulum. The use of this approach for smaller subcellular compartments (mitochondria, lysosomes, vesicles, etc.) has not been evaluated systematically so far, and it may require isolation of organelles to obtain ion intensities above the limit of detection. The described ionomics assay is applicable in a LTP mode (6-well plate). Another limitation of the assay is that the ICP-MS is a relatively expensive equipment and therefore not available in the vast majority of labs.

### Binding Assays

Binding assays are based on assessing direct interactions between the compound and the target. These assays can be useful to find binders of SLCs, not necessarily only compounds acting as inhibitors. Such binders can function as pharmacological chaperones, potentiate or prevent PPIs (Passioura et al., 2018), or can be modified into chemical chimeras such as PROTACs (Schreiber, 2019).

Using approaches of chemical proteomics, binding assays can be focused on compound (chemical-centric) or protein (target-centric), depending on the nature of the bait. Chemical-centric methods, recently reviewed in (Robers et al., 2020), have been used for many years to deconvolute targets from phenotypic screens, or to profile off-target effects of compounds on a proteome level. Methods such as photoaffinity labelling and *Click* chemistry, or thermal proteome profiling (TPP), are capable of reporting low affinity and less abundant interactions – in principle including a drug and its transporter (Parker and Pratt, 2020). Thus, these methods may be suitable starting points to screen for the SLC responsible for transport of an investigated compound. However, MS-based proteome-wide profiling frequently exhibits a bias towards soluble proteins, and thus interactions with SLCs may be underrepresented, though examples of their use to deconvolute a SLC as a direct target of drugs exist (Parker et al., 2017b).

Target-centric binding assays are in general suitable for HTP chemical screening (Alexandrov et al., 2008; Hall et al., 2016) and especially in combination with technologies such as DNA-encoded libraries can screen very large chemical libraries (Passioura et al., 2018). However, these assays frequently require purified protein. Since protein purification for membrane proteins with several transmembrane domains is in general considered challenging (Wang W. W. et al., 2019), we focus on the cellular thermal shift assay, which has been recently optimized for SLCs and does not require purified protein (Hashimoto et al., 2018).

#### Thermal Shift Assay

**FIGURE 5:**
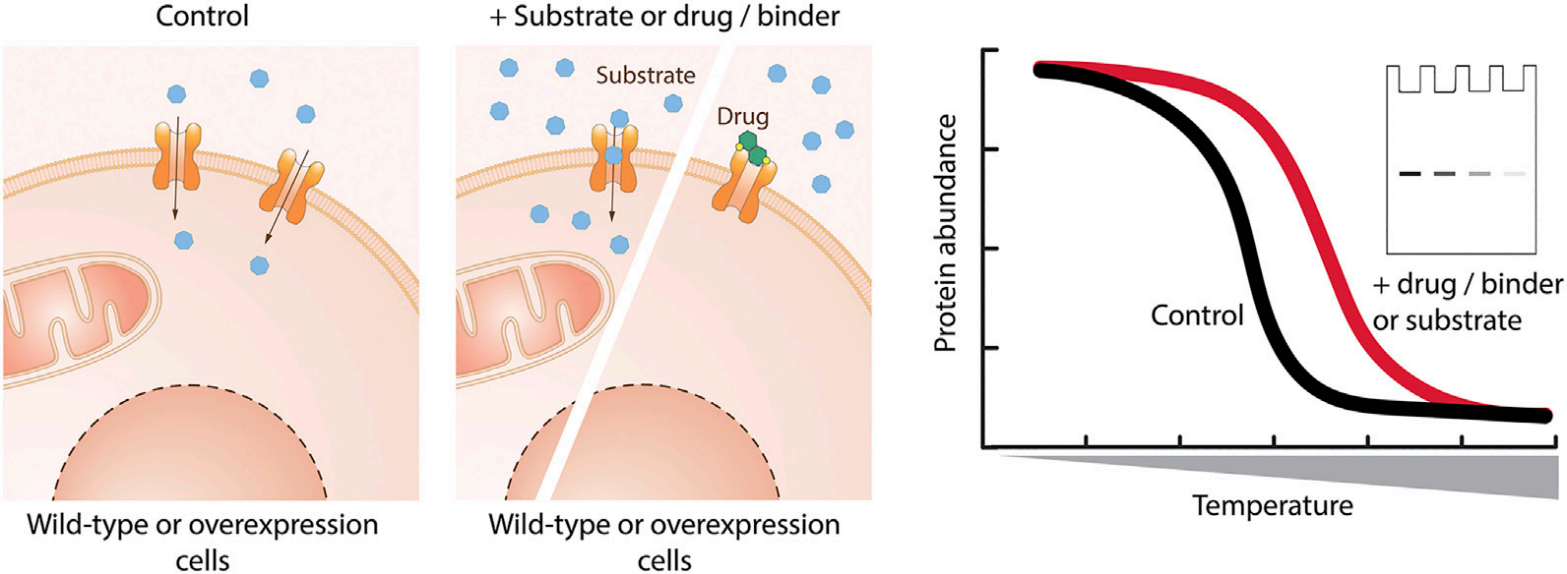
Cellular binding assay based on thermal shift. Cells are incubated with the molecule of interest, lysed, and exposed to increasing temperature. The remaining protein in native conformation is quantified by western blotting or using reporters. Binding of a small molecule stabilizes the protein of interest and leads to a shift in the melting temperature of the protein of interest.

The thermal shift assay (TSA) using cells is based on the behavior of a protein exposed to increasing temperatures (Martinez Molina et al., 2013). Upon reaching a certain temperature (melting temperature – Tm), the thermodynamic stability of the protein fold is disrupted, resulting in protein unfolding and aggregation with other unfolded proteins. Interaction of the protein with a small molecule can result in partial thermal stabilization, and thus in a shift in Tm (Figure 5). In this way, direct protein-ligand engagement can be assayed. In comparison to the *in vitro* TSA (Alexandrov et al., 2008; King et al., 2016; Tavoulari et al., 2019), the cellular TSA assesses protein-ligand interactions in a cellular environment and does not require purified protein. While the TSA was originally established as a method to determine a drug-target engagement, in recent years the cellular TSA is emerging also as an assay for primary screening (Shaw et al., 2019).

##### Technical Requirements and Level of Throughput

For performing a cellular TSA experiment, a source of heating (such as thermoblock or PCR cycler) and a readout discriminating native from aggregated protein are necessary. The choice of readout determines the assay throughput. The most widely used method with Western blotting (WB) as a readout (Martinez Molina et al., 2013) can test only limited compound-target combinations but technologies such as AlphaScreen (Almqvist et al., 2016) or split reporters can enable screening in HTP (reviewed in Henderson et al., 2019). Using MS as a readout, the method can be applied to study target engagement of a single compound on proteome level (Huber et al., 2015; Reinhard et al., 2015).

##### Experimental Setup

Since the method was originally introduced for cytoplasmic proteins (Jafari et al., 2014), modifications were necessary for membrane proteins (Hashimoto et al., 2018). Typically, compounds are incubated either with intact cells or cell lysates. The use of intact cells accounts for membrane crossing or metabolic modifications of the compound. Next, samples are aliquoted and exposed to heating. Lysing the cells prior to a heating step could facilitate easier aggregation of membrane proteins after melting, however some studies with membrane protein lysed the cells only after the heating step (Huber et al., 2015; McMillan et al., 2018; Kawatkar et al., 2019). To avoid resolubilization of aggregates, a mild detergent should be used (Reinhard et al., 2015). Finally, the remaining protein in native conformation is quantified in each sample. The most commonly used technique is to remove aggregates with centrifugation and quantify the native protein by WB, but readouts employing reporters can specifically distinguish native protein, and thus the removal of aggregates is not necessary (Martinez et al., 2018). Dose dependency can be confirmed via an isothermal dose-response fingerprint (ITDRF_CETSA_) experiment where the sample is treated with several ligand concentrations at constant temperature (Martinez Molina et al., 2013).

Length of compound incubation, compound concentration, sample volume, cell density, heating duration, and the efficiency of native–aggregated protein discrimination should all be optimized first. If available, a potent and selective ligand, such as a specific inhibitor, can be used to determine the possible degree of Tm shift. However, similarly potent inhibitors targeting the same protein can have a different degree of Tm shift (Kawatkar et al., 2019). Although the most widely used heating duration is 3 min, longer heating duration could result in a better Z’ factor and thus be beneficial for HTP screening (Martinez et al., 2018).

##### Suitable Solute Carrier Families

A proof-of-principle study showed thermal stabilization of members of SLC1 and SLC16 families upon treatment with available inhibitors, and in the case of SLC16 also stabilization with substrate (Hashimoto et al., 2018). Although to our knowledge only few other studies use the cellular TSA to target SLCs, namely SLC2 family (McMillan et al., 2018; Reckzeh et al., 2019), a number of studies have applied the cellular TSA for transmembrane proteins (Huber et al., 2015; Reinhard et al., 2015; Kawatkar et al., 2019) and SLCs were also detected in TPP studies (Reinhard et al., 2015), demonstrating the potential to apply the method more broadly.

##### Assay Advantages, Limitations and Approximate Costs

Advantages are that the TSA is probing the direct interaction of a target with a compound, its versatility and that it can use lysate, intact cells, and even whole tissue (Martinez Molina et al., 2013). However, the major limitation is that not all compounds binding the protein will shift the Tm for reasons like insufficient stabilization of the structure or loss of the interaction between protein and compound due to high, non-physiological temperatures. Thus, while the method is relatively resistant to false-positive results, false-negative results can occur. The Drug Affinity Responsive Target Stability (DARTS) assay represents an alternative assay technology similar to the TSA that can assess drug-target engagement for SLC inhibitors (Lomenick et al., 2009; Benjamin et al., 2018). The costs of running a LTP cellular TSA are basically equal to the costs of running a WB experiment, while costs of a HTP TSA depend on the readout and thus on reagents.

### Functional Assays

In contrast to substrate uptake assays, functional assays do not assess the transporter activity directly, but are measuring the secondary effects caused by SLC driven transport, such as changes in membrane potential or intracellular pH. While employing functional assays to poorly characterized SLCs may be challenging, implementation for SLCs which are sufficiently characterized may be relatively easy, and many of these assays can be also easily optimized for HTP. Functional assays should be followed-up with a counter-screening campaign, to confirm that the primary screening hits are truly connected to SLC mediated transport.

#### Fluorescent Dyes

**FIGURE 6:**
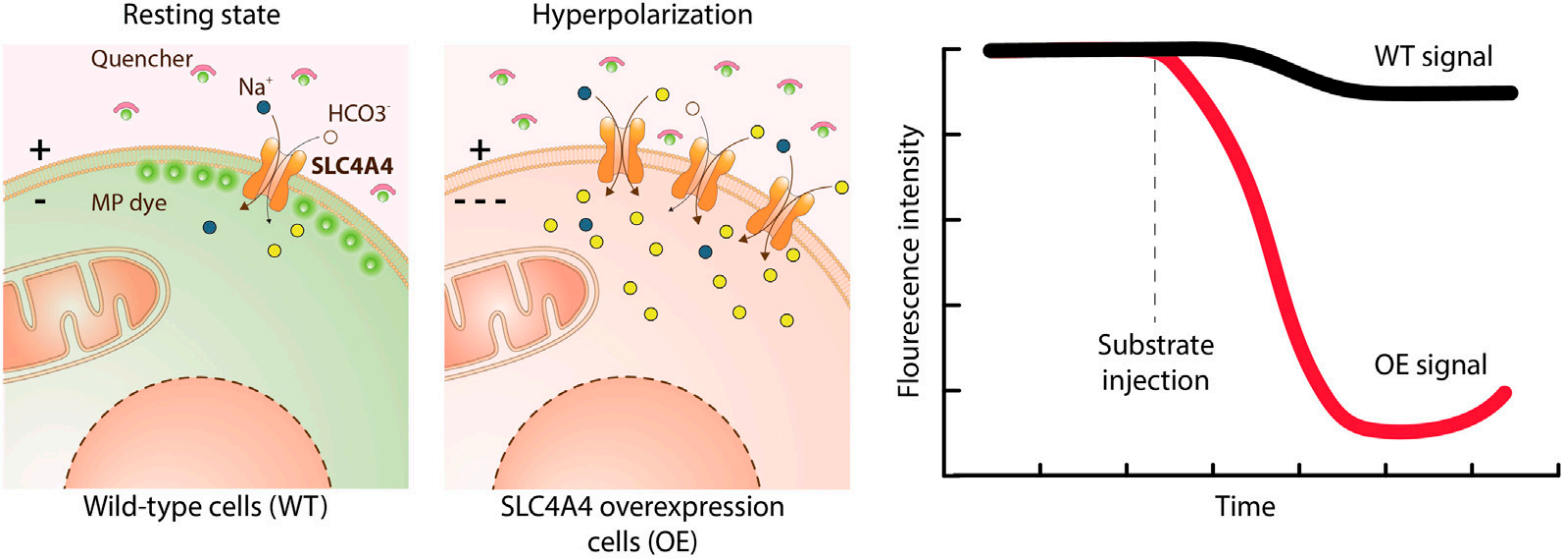
Transport assay using a membrane potential dye. This assay uses a chemical dye to detect changes in the membrane potential (MP) caused by the ions transported by an electrogenic SLC. The dye coupled to a quencher is added to the medium. In the resting state, some dye enters the cell causing a fluorescent intensity that serves as a reference. Upon membrane hyperpolarization the dye does not penetrate in the cells and remains attached to the quencher, resulting in a fluorescence decrease. Upon depolarization of the membrane the dye detaches from the quencher and penetrates into the cells, eliciting a signal increase. Overexpression of SLC4A4 (a 1:Na^+^/3:HCO^3−^ co-transporter) and addition of its substrates leads to hyperpolarization and a decrease in fluorescence intensity over time compared to wild-type cells.

A number of functional assays is based on fluorescent dyes, which are either sensitive to changes in membrane potential or in ion concentrations (Yu et al., 2015). Membrane potential sensitive dyes measure changes of charges across the cell membrane. FLIPR membrane potential dye (Molecular Devices) is a lipophilic, anionic, bis-oxonol dye able to cross the plasma membrane and to measure voltage changes by its potential-dependent accumulation and redistribution (Wolff et al., 2003) (Figure 6). When the cells are depolarized, the dye enters the cells, causing an increase in fluorescent signal, conversely, cell hyperpolarization results in dye exit and decreased fluorescence. Ion sensitive dyes measure changes in the concentration of a specific ion, such as calcium, sodium, potassium or changes in pH. Several calcium-sensitive dyes are available, with different calcium affinities and different excitation/emission spectra. Among these, Fluo-8 dyes were developed to improve other dyes (e.g. Fluo-3, Fluo-4) in terms of loading and brightness. Among pH sensitive dyes, the most used is 2′,7′-bis(2-carboxyethyl)-5,6-carboxyfluorescein (BCECF-AM), a non-charged indicator that rapidly diffuses inside the cell, where intracellular esterases cleave the ester bond releasing BCECF, which fluoresces according to the intracellular pH (Ozkan and Mutharasan, 2002; Benjamin et al., 2005). Sodium sensitive dyes are used to detect changes in Na^+^ concentrations. Two of the most frequently used are Asante Natrium Green and CoroNa (Iamshanova et al., 2016). In contrast to Ca^2+^ and pH sensitive dyes, Na^+^ sensitive dyes are not well-suited for HTP screening due to low sensitivity and a poor signal-to-background ratio for SLC targets (Yu et al., 2015). Potassium transport is frequently studied though exploiting thallium as a surrogate ion for potassium. Some examples are the FLIPR Potassium Assay kit (Molecular Devices) and the FluxOR Potassium Ion Channel Assay (Thermo Fisher). The increase in cytosolic thallium is detected using the thallium-sensitive dye indicator (Weaver et al., 2004).

##### Technical Requirements and Level of Throughput

Detection of fluorescent indicator dyes is achieved by means of a fluorescence plate reader (such as Fluorescent Imaging Plate Reader (FLIPR) or Hamamatsu FDSS) able to excite the probe and to read its emission. This instrumentation together with the characteristics of the dyes enable the HTP of the assay. Alternatively, fluorescence can be assessed by microscopy or flow cytometry.

##### Experimental Setup

The cell line with the expression of the SLC of interest together with an adequate control cell line (mock control that does not express the target, not-induced if the target is overexpressed with an inducible system or knock-out cell line) is loaded with the fluorescent dye in a suitable buffer for a period that typically ranges from 10 min to 1 h. Then, the solution is exchanged to an appropriate assay buffer containing test compounds (e.g. drug candidates). Next the buffer containing a transporter substrate is added and changes in fluorescence are measured. By fitting the concentration of the tested compound against the changes in fluorescence, the EC_50_ value is obtained.

##### Suitable Solute Carrier Families

The membrane potential dye has been used to study electrogenic transporters from families SLC1 (Jensen and Bräuner-Osborne, 2004) and SLC6 (Benjamin et al., 2005; Danthi et al., 2019). BCECF dye has been used to study SLCs from SLC9 (Windler et al., 2018), SLC16 (Contreras-Baeza et al., 2019) and SLC12 families (Reynolds et al., 2007). Potassium transport was measured with the thallium dye (as a surrogate) for SLC12 family (Carmosino et al., 2013) and with the sodium indicator Asante Natrium Green for SLC4 and SLC12 families (Noor et al., 2018).

##### Assay Advantages, Limitations and Approximate Costs

The main limitation in the use of dyes is that a cell loading step is required, with the consequent risk of affecting cell physiology. Nevertheless, most of the dyes are very easy to load and require a single incubation step without washing the cell monolayer (Wolff et al., 2003), which results in a rapid and HTP assay. In addition, these assays are flexible and have low temporal resolution, given the dyes' fast responses. Finally, the use of probes brings along with it some elevated costs.

#### Electrophysiology

Since its discovery by Neher and Sakmann (Neher and Sakmann, 1976), the patch clamp technique has been widely used to study membrane electrical activity and the underlying ion currents in excitable cells. Today patch clamp is still recognized as the golden standard technique to study voltage- and ligand-gated ion channels, as well as mechanosensitive, transient receptor potential (TRP) channels and electrogenic proteins, such as pumps or transporters (Brown and Greenberg, 2016). Compared to surrogate techniques, such as fluorescence or luminescence assays, patch clamp allows not only to identify active molecules on the target of interest, but it also provides information about the mechanism of action of a compound. Due to its direct measurement of net charge fluxes across the membrane, patch clamp is a very powerful tool for mechanistic studies.

##### Technical Requirements and Level of Throughput

Patch clamp requires one skilled person to run a so-called “electrophysiology setup”. The basic version is composed of an inverted microscope, an operational amplifier and a digital-analogic transducer coupled with a computer for data collection and analysis. Usually, a Faraday cage is included for electrical isolation and a fluidic perfusion system is in place to apply compounds diluted in physiological saline solutions (Rubaiy, 2017). Since the technique is versatile, any laboratory currently equipped with the setup can extend this approach to study SLCs without major changes to the protocols already in place. The high informativity is given at the cost of the intrinsic LTP.

##### Experimental Setup

The day before the experiment SLC-expressing cells are seeded as single isolated cells on coated glass coverslips. On the day of experiment the coverslips with cells are placed in the recording chamber of the inverted microscope equipped with the headstage of the operational amplifier to run the patch clamp experiment. A glass micropipette is filled with the solution mimicking the cytosolic environment, and firmly stabilized on the headstage of a micromanipulator. The tip of the micropipette is carefully attached to the cell membrane. To form an electrical seal between the micropipette and cell membrane, a constant negative pressure is applied. The membrane is ruptured by a sudden pulse of negative pressure or by brief applications of currents. Afterwards, the voltage clamp configuration is used to modulate the cell membrane potential by applying voltage waveforms specifically designed to favor the activation of the SLC under investigation.

Typically, a perfusion system is integrated in the manual patch clamp setup to apply inhibitors, activators or substrates directly on the patched cell. If such a system is not available, compounds can be directly applied by pipetting small amounts of solution in the recording chamber.

The changes in the membrane currents upon application of substrates/inhibitors are recorded in real-time and analyzed offline, with the major advantage being the internal normalization control for each application of a given compound since transmembrane current is measured before and after.

##### Suitable Solute Carrier Families

The SLC of interest needs to be electrogenic (Supplementary Table S1) and expressed in sufficient amount at the plasma membrane. Patch clamp was used for example to validate the effects of two molecules (SEA0400 and KB-R9743) now recognized as reference SLC8A1 inhibitors (Elias et al., 2001; Lee et al., 2004).

##### Assay Advantages, Limitations and Approximate Costs

The main advantage is the high time resolution and the accuracy of the readout signal, allowing direct monitoring of electrogenic protein activity and their modulation by compounds in real-time. Single cell analysis of a stable clone provides information about homogeneity of the cell line, i.e. the percentage of cells functionally expressing the SLC of interest. A disadvantage for its use for SLCs may be that the net charge caused by electrogenic transport is much lower compared to the opening of an ion channel. The amplitude of recorded signals may not be high enough to allow a dose-response experiment and different techniques are required for a full pharmacological characterization, unless the SLC is expressed at very high levels.

#### Solid-Supported Membrane Based Electrophysiology and Surface Electrogenic Event Reader Technology

**FIGURE 7:**
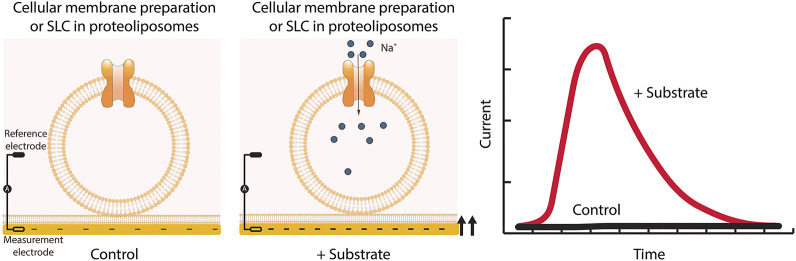
SSM-based electrophysiology applied to SLCs. Membrane preparations from cells overexpressing the SLC of interest are applied to the sensor and together form a capacitively coupled membrane system. Therefore, charge translocation at the protein containing membrane can be detected via the SSM. After addition of the SLC substrate, changes in membrane potential are recorded. Only transient currents are measured, and the peak current represents the maximum speed of the transport.

Solid-supported membrane (SSM)-based electrophysiology was especially developed for the measurement of transporters such as SLCs, which are difficult to investigate using conventional electrophysiology (Nanion Technologies Munich, 2021; Schulz et al., 2008; Bazzone et al., 2013, 2017). The methodology differs from conventional electrophysiology. Instead of living cells, the methodology uses diverse native or artificial membrane vesicles, such as reconstituted protein in proteoliposomes or membrane preparations from organelles, cells or tissue samples (Nanion Technologies Munich, 2021; Geibel et al., 2006; Bazzone et al., 2013, 2017). The membrane sample is added to a SSM, which consists of a lipid monolayer on top of a thiolated gold coated sensor chip. This leads to the stable adsorption of the added membranes to the SSM and the formation of a capacitively coupled membrane. The experiment starts in the presence of buffer lacking the SLC substrate. During the experiment the buffer is exchanged for a solution containing the SLC substrate. The substrate gradient established by fast solution exchange is the main driving force and the transport of charged substrates or ions into the liposomes or vesicles generates a membrane potential. The potential is detected via capacitive coupling between the membrane and the SSM on the gold layer of the sensor. As soon as the membrane potential equals the chemical driving force, the transport process comes to a halt. The *surface electrogenic event reader* technology (*SURFE* ^*2*^ *R*) (Nanion Technologies Munich, 2021) employs SSM-based electrophysiology and allows the measurement of up to 10^9^ transporters at the same time to yield the best signal to noise ratio (Figure 7).

##### Technical Requirements and Level of Throughput

SSM-based electrophysiology requires a *SURFE* ^*2*^ *R* instrumentation, which is available as a *SURFE* ^*2*^ *R N1* for LTP assays, or as a *SURFE* ^*2*^ *R 96 SE* enabling HTP and automatization in a 96-well plate like format (96 sensors in parallel) (Nanion Technologies Munich, 2021). Each membrane containing the protein of interest is suitable for sample preparation and for measurements using SSM-based electrophysiology, but nevertheless a high protein density and purity can compensate for low turnover and low electrogenicity (Nanion Technologies Munich, 2021; Bazzone et al., 2013, 2017). Normally, the transporter of interest is either recombinantly overexpressed or used from native tissue including different organisms. Commonly used expression systems range from bacteria to eukaryotic cell lines. Also, cell-free expression systems have been used to assay transporter function with SSM-based electrophysiology, where protein is purified, followed by reconstitution into liposomes of ∼100 nm in diameter at high protein densities (Barthmes et al., 2016; Bazzone et al., 2017). An advantage of reconstituted samples is the possibility to vary the membrane composition of the sample which can affect the protein function or ion gradient stabilities (Bazzone et al., 2013, 2017). Due to its mechanical robustness, SSM-based electrophysiology has a high potential for screening applications, allowing determination of the dose dependence of 100 compounds in less than 30 min (Nanion Technologies Munich, 2021; Bazzone et al., 2013, 2017). A *SURFE* ^*2*^ *R 96SE* system (Nanion Technologies Munich, 2021) allows the recording of 96 wells in parallel and the automatization of experimental workflows including sensor preparation, data analysis and export, and practically results in the measurement of six 96-well plates per day (Nanion Technologies Munich, 2021).

##### Experimental Setup

For the laboratory setup, a detailed protocol performing experiments was published by Bazzone et al. in 2013 (Bazzone et al., 2013). Sensor preparation includes the thiolization of the sensor surface, the assembly of the lipid layer, and finally the application of membranes to the sensor (Nanion Technologies Munich, 2021). The protein containing membrane and the SSM will form a capacitively coupled membrane system and therefore, charge translocation at the protein containing membrane can be detected by capacitive coupling via the SSM. Upon substrate addition, transient currents are recorded, whereas the peak current represents the maximum speed of the transport. Due to the high stability of the SSM, up to one hundred sequential measurements can be performed on the same sample and allow the determination of parameters such as EC_50_ or IC_50_ (Nanion Technologies Munich, 2021). SSM-based electrophysiological experiments only require 0.1 – 1 µg protein per sensor (Nanion Technologies Munich, 2021; Bazzone et al., 2013, 2017).

##### Suitable Solute Carrier Families

The method is suitable for the detection of any kind of reaction associated with a charge displacement or with the change in water accessibility close to a charge (electrogenic transporters listed in Supplementary Table S1). The SLC families which were measured using this method are for example SLC1, SLC6, SLC8, SLC15 or SLCO (Geibel et al., 2006; Bazzone et al., 2017; Gerbeth-Kreul et al., 2021; Nanion Technologies Munich, 2021). In addition, since SSM-based electrophysiology has been used to assess the function of mitochondrial proteins, this technique may be applied to intracellular SLCs (Watzke et al., 2010).

##### Assay Advantages, Limitations and Approximate Costs

The technology allows real-time data acquisition with a high time resolution and a high signal amplification compared to conventional patch-clamp (Nanion Technologies Munich, 2021). SSM-based electrophysiology additionally allows to resolve fast binding kinetics and EC_50_ or IC_50_ determination in a HTP manner (Nanion Technologies Munich, 2021).

In contrast to patch clamp and voltage clamp techniques, SSM-based electrophysiology cannot be used to apply a membrane potential (Bazzone et al., 2013). Transporter characterization is therefore restricted to transport modes which do not rely on a membrane potential (Bazzone et al., 2013). In general, SSM-based electrophysiology has no limitations concerning the type of the transporter, but voltage clamp or patch clamp methods can have advantages, if intracellular components like binding proteins are required for protein functionality (Bazzone et al., 2013, 2017). Limitations can arise, if solution exchange creates large artifact currents which happens when the substrate interacts strongly with the SSM like in the case of lipophilic compounds (Bazzone et al., 2013).

Costs per data point are dependent on the assay protocol, especially how many activations, concentrations of compounds or conditions are tested in one well/sensor.

Overall, SSM-based electrophysiology is an ideal methodology in cases where conventional electrophysiology cannot be applied and is also attractive for screening applications in drug discovery especially because of its robustness and its potential for automation (Geibel et al., 2006; Nanion Technologies Munich, 2021).

#### Solute Carrier-G Protein Coupled Receptor Coupling

**FIGURE 8:**
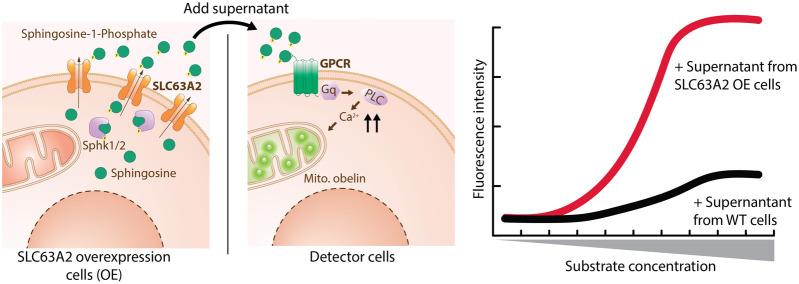
SLC-GPCR coupling assay applied to SLC63A2. Sphingosine is phosphorylated by Sphk1/2 and exported by SLC63A2 OE cells through SLC63A2 into medium. Supernatant from these cells is then applied to detector cells, which stably express a S1P specific GPCR and the Ca^2+^ reporter Obelin. Activation of the GPCR as a surrogate readout for SLC63A2 transport of S1P is quantified by the increase of reporter fluorescence.

An SLC-GPCR coupling assay is based on detecting the SLC substrate via GPCR engagement. The assay consists of two parts: first, the SLC is stimulated to export its substrate, and second, a GPCR which recognizes the substrate as a ligand is used for detection (Figure 8).

##### Technical Requirements and Level of Throughput

The hardware requirements for an SLC-GPCR coupling assay are a fluorescence or luminescence microtiter plate reader which ideally allows for kinetic measurements. GPCR activation typically leads to a change in the intracellular calcium or cyclic adenosine monophosphate (cAMP) concentrations, or to altered gene expression. These events can be easily detected by using a typical “GPCR toolbox” consisting mainly of fluorescent dyes and genetically encoded luminescent biosensors (Thomsen et al., 2005; Zhang and Xie, 2012). In brief, calcium responsive fluorescent dyes (e.g. Fluo-8) or photoproteins (e.g. aequorin) can be used to detect increases in intracellular calcium (Ma et al., 2017). Changes in cAMP levels can be detected e.g. via a cAMP-responsive luciferase (Fan et al., 2008), or proximity-based homogenous assays relying on cAMP-antibodies (Williams, 2004). Alterations in gene expression are typically monitored by inserting a reporter, e.g. a luciferase, into the respective genomic locus. All these assays can be run in 384-well-plates which allows for HTP.

##### Experimental Setup

For assay development, two cell lines are generated: one expressing the SLC and the other cell line expressing the corresponding GPCR. In a first step, the GPCR assay is independently optimized using the GPCR ligand (i.e. the SLC substrate) from an external source, as well as known inhibitors of the GPCR to verify sensitive detection of GPCR activation, inhibition, and desensitization. In a second step, the supernatant of the SLC-expressing cell line can be used for GPCR activation. At this stage, different SLC stimulation parameters can be evaluated, and clonal selection can be performed. It is also conceivable to express both the SLC and the GPCR in a single cell line. Specifically, for assaying SLC59A2 (MFSD2B) and SLC63A2 (Spinster2, SPNS2), we generated cell lines which stably express SLC59A2 or SLC63A2 + SPHK1 (sphingosine kinase 1) and feed these cells with (unlabeled) sphingosine for several hours. Intracellular sphingosine kinases phosphorylate the pre-substrate to S1P, which is exported by the SLC. Transferring the SLC cells’ supernatant to cells co-expressing S1P3 (a Gq coupled S1P receptor) as well as the calcium photoprotein obelin results in a luminescent calcium response.

In order to make the assay more amenable to HTP, we adapted it to a desensitized format: Continuous agonist stimulation eventually leads to GPCR desensitization, inactivation or internalization (Siehler and Guerini, 2006; Rajagopal and Shenoy, 2018). Thus, instead of detecting the SLC substrate (S1P) as an agonist, it can also be detected as a functional antagonist when applying a second agonist challenge using the substrate (S1P) from an external source. A previously desensitized GPCR will remain “silent”, while not previously stimulated GPCRs will show an agonistic signal. Once optimized, we adapted the desensitized format to a co-culture set-up where S1P3 and SLC cells are seeded into the same well, to eliminate the need for supernatant transfer.

##### Suitable Solute Carrier Families

We have successfully applied this strategy to set up assays for the two sphingosine-1-phosphate (S1P) transporters SLC63A2 and SLC59A2. Previously described assays for these transporters were low in throughput because they involved laborious sample preparation followed by TLC (thin layer chromatography) or HPLC (high performance liquid chromatography) detection of radiolabeled or fluorescent S1P (Kawahara et al., 2009; Hisano et al., 2012; Vu et al., 2017; Kobayashi et al., 2018). This approach is applicable to any other SLC which can export a GPCR ligand into the extracellular medium.

##### Assay Advantages, Limitations and Approximate Costs

SLC-GPCR coupling involves several steps which need optimization and pose confounding factors that may lead to false positive/negative hits. For HTP screening, this approach is therefore most suitable for SLCs which cannot be assayed with more straightforward options such as membrane potential or pH-sensitive dyes, a fluorescent surrogate substrate, or a substrate-specific biosensor. On the other hand, GPCR coupling poses a highly specific and sensitive tool for substrate detection. Therefore, SLC-GPCR coupling can be a valuable downstream or orthogonal assay for hit validation. The cost of an SLC-GPCR coupling assay heavily depends on the GPCR readout strategy. In the S1P transporter assay described above, the cost is low with coelenterazine (obelin’s luminophore) being the most expensive reagent per well.

#### Label-free Impedance-Based Assay

Label-free cell-based assays have emerged in recent years as a versatile platform to monitor changes in cellular properties such as adhesion, proliferation, growth and morphology (Xi et al., 2008). Several platforms, e.g. optical and impedance-based technologies, have been used to develop such assays as drug discovery tools for protein classes like GPCRs (Yu et al., 2006). In principle, activation of a GPCR on living adherent cells generates a whole-cell response dependent on coupling to intracellular signaling pathways and cellular background, leading to temporal changes in cell morphology which are detected in real-time (Scott and Peters, 2010). Impedance-based assays, e.g. xCELLigence (Doornbos and Heitman, 2019), have been extensively used for functional characterization of GPCR agonists, antagonists, and allosteric modulators (Doornbos et al., 2018). Since SLC substrates can act as GPCR ligands, this technology is suitable for assaying SLCs. The resulting assay, in which SLC activity or inhibition can be measured via GPCR activation, was termed the “transport activity through transport activation” (TRACT) assay (Sijben et al., 2021).

##### Technical Requirements and Level of Throughput

Requirements to run xCELLigence experiments are a general cell culture facility, temperature and CO_2_ controlled environment (e.g. cell culture incubator), an xCELLigence real-time cell analysis (RTCA) instrument and E-plates, which are the main consumable of this application. The xCELLigence RTCA system has multiple plate configurations ranging from 16 to 96 wells up to 384 wells which are amenable to HTP screening (Halai and Cooper, 2012). To allow detection of SLC activity, a (preferably adherent) cell line is required with heterologous or endogenous expression of both the SLC and a concomitant GPCR.

##### Experimental Setup

**FIGURE 9:**
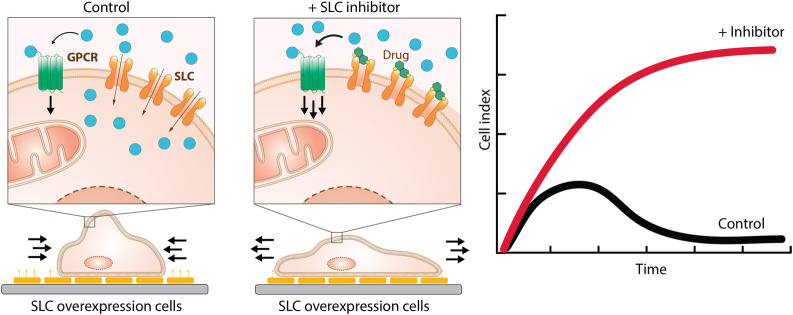
TRACT assay. Activation of a GPCR leads to changes in cellular morphology which can be quantified using the xCELLigence system. Exogenous addition of a SLC substrate which is at the same time a GPCR ligand to cells expressing both the SLC and the GPCR will lead to partial uptake and activation of the GPCR, measured by morphological changes with the xCELLigence real-time cell analysis (RTCA) instrument. Overexpression of the SLC leads to increased uptake of the substrate, which attenuates the GPCR-mediated cell response. When SLC transport is blocked by an inhibitor, the extracellular concentration of the SLC substrate/GPCR ligand is increased which leads to augmented activation of the GPCR and an enhanced cell response.

Reports on label-free cell-based transporter assays have been limited (Wong et al., 2012). Recently, a label-free impedance-based assay was developed using xCELLigence to assess functional activity of SLC29A1 (equilibrative nucleoside transporter 1 or ENT1) in living cells (Vlachodimou et al., 2019). Here, activation of adenosine receptors (ARs) by adenosine, a SLC29A1 substrate/AR agonist, is used as a readout. The assay is based on the hypothesis that active SLC29A1 mediates influx of adenosine when extracellular concentrations are higher than cytosolic adenosine, thereby controlling the tone and magnitude of adenosine-mediated signaling events. Upon addition of exogenous adenosine to cells that endogenously express both SLC29A1 and ARs, adenosine is partially taken up via SLC29A1, while the remaining extracellular adenosine is able to activate ARs expressed on the cell membrane. When SLC29A1 transport is blocked by an inhibitor, the extracellular concentration of exogenous adenosine is increased which leads to augmented activation of ARs resulting in an enhanced cell response. This provides an assay window which has been used to characterize inhibitory potency (Vlachodimou et al., 2019) and binding kinetics (Vlachodimou et al., 2020) of SLC29A1 inhibitors. More recently, this assay principle was validated for the human dopamine transporter (DAT, SLC6A3) in two cell lines with heterologous expression of DAT (Figure 9) (Sijben et al., 2021).

##### Suitable Solute Carrier Families

SLCs that are suitable for assessment with a TRACT assay should have a known substrate ascribed to them, meaning that orphan transporters are not amenable. So far, TRACT assays have been developed for SLC29A1 and SLC6A3 using the xCELLigence technology. In theory, any SLC that transports a substrate which at the same time is an agonist for a membrane-bound GPCR is admissible for this assay. Examples of these are SLCs for monoamine neurotransmitters (e.g. SLC6A2, SLC6A4), glutamate (SLC1) (Doornbos et al., 2018), carboxylic acids (SLC16), and fatty acids (SLC27). To further widen the scope, any SLC that is involved in or influences a process that leads to detectable changes in cell morphology could potentially be assessed (Wang W. W. et al., 2019). However, this remains to be demonstrated experimentally.

##### Assay Advantages, Limitations and Approximate Costs

Label-free cell-based assays detect whole-cell responses that are essentially an accumulation of all intracellular signaling events resulting from a perturbation. This allows the researcher to capture comprehensive information in real-time without the use of non-physiological labels and invasive methods. The cumulative signal may also be perceived as a disadvantage of this approach as it produces a “black box” readout, which warrants thorough signal validation during assay development. Additionally, compounds inducing off-target effects that mask or amplify observed cell responses can result in false positives or false negatives, which are mitigated with appropriate counter screens. Main costs for running xCELLigence assays come down to the E-plate consumables. Some protocols describe the reuse of E-plates which provides perspective to reduce overall assay costs (Stefanowicz-Hajduk et al., 2016).

#### Solute Carrier Coupling to Nuclear Hormone Receptor

Human nuclear hormone receptors (Maglich et al., 2001) are ligand dependent transcription factors, which activate or repress the transcription of genes after binding the corresponding hormone. 48 members of this protein family are known. While the majority are still orphans, the associated ligands for some of those or their precursors are SLC substrates.

An example are steroid sulfates, transported by SLC10A6 (sodium dependent organic anion transporter or SOAT), but also by members of the SLCO (OATP) and SLC22 (OAT) families (Pizzagalli et al., 2003; Ugele et al., 2003). As a result of transporter activity, the intracellular hormone precursor concentration is increased. After the sulfate has been cleaved off by steroid sulfatase (Reed et al., 2005), the agonism of the active steroid is measured at the corresponding nuclear receptor.

**FIGURE 10:**
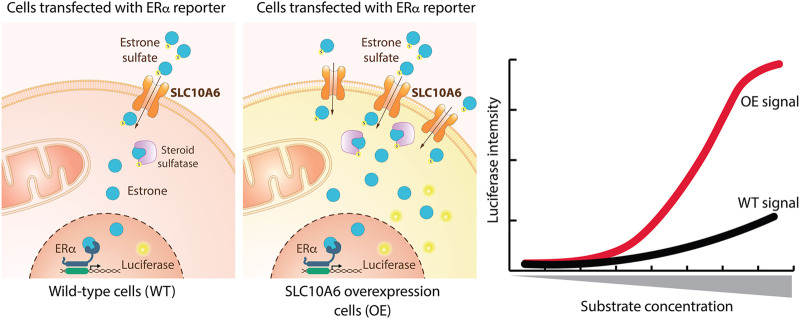
SLC coupling to nuclear hormone receptor applied to SLC10A6. A cell line expressing a reporter plasmid combining a response element of the estrogen receptor alpha (ERα) and a luciferase encoding gene is treated with estrone sulfate, which is imported to the cytoplasm by SLC10A6 and cleaved by the steroid sulfatase. The product estrone then binds to the estrone-responsive element and activates luciferase expression from the reporter. Inducible overexpression of SLC10A6 leads to increased uptake of estrone sulfate and therefore increased luciferase intensity.

There are two established methods to quantify nuclear receptor activity in cellular assays (Schulman and Heyman, 2004; Pinne and Raucy, 2014). The first protocol is a transactivation assay based on the transient or stable expression of the nuclear hormone receptor combined with a plasmid carrying a response element for this receptor in front of a reporter gene like β-galactosidase or luciferase. The second method employs a fusion between the ligand binding domain of the nuclear receptor and the yeast GAL4 protein (yeast galactose metabolic genes inducing transcription factor 4), in combination with a vector carrying the GAL4 UAS (upstream activating sequence) in front of a reporter enzyme (Figure 10).

##### Technical Requirements and Level of Throughput

The assay needs to be performed in a cell culture lab and requires a suitable microtiter plate reader for luminescence or absorbance measurements. Both assay systems, the protocol using the full-length nuclear receptor as well as the GAL4 fusion method can be adapted to a HTP format. The GAL4 fusion protocol shows in general higher signal windows.

##### Experimental Setup

In order to establish a transporter assay cell line, the expression constructs for the SLC, the corresponding nuclear hormone receptor and its reporter plasmid are transfected into a suitable mammalian host cell line. An inducible expression of the target SLC will enable the analysis of the contribution from the endogenous SLCs in the host cell line to the total hormone uptake. The assay starts by incubating the test compounds with the cells, then the SLC substrate is added for a given time, followed by its removal and an additional incubation time for the cells to express the reporter. Optimization of SLC substrate concentration and incubation time can improve assay performance.

##### Suitable Solute Carrier Families

In principle all SLCs for which nuclear receptor agonists or their precursors are substrates can be assayed in this format based on steroid sulfates and corresponding nuclear hormone receptors, such as SLC10A6, members of the SLCO and SLC22A families. Thyroid hormone transporters like SLC16A2 and SLC16A10 might also be amenable for this assay type (Visser et al., 2011).

##### Assay Advantages, Limitations and Approximate Costs

An advantage of this assay method is the use of unmodified, natural SLC substrates and its independence of electrogenicity or cotransport of certain ions. An important limitation for some substrates is redundancy, especially when respective SLCs are endogenously expressed in the host cell line. The costs of running the assay are low.

#### Phenotypic Assays

**FIGURE 11:**
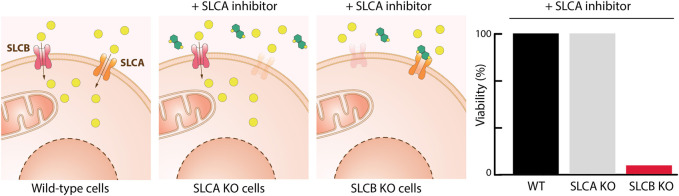
Phenotypic assay based on synthetic lethality. WT cells are expressing SLCA and SLCB which are two transporters with a strong negative genetic interaction. KO of SLCA results in cells dependent on SLCB and *vice versa*. Therefore, selective inhibitors of SLCA kill only SLCB KO cells.

Phenotypic assays (PAs) rely on a “visible” or observable read-out as a proxy for a biological/biochemical function. PAs useful for transporter research typically involve cell lines, although employing a variety of models, from yeast to fish, is feasible in principle. Among the cell line-based assays one can distinguish phenotypes that are based on inherent cellular properties, such as fitness/survival, cellular differentiation, or adhesion. The key principle relies on the ability of genetics to demonstrate that a particular cellular feature is dependent on the function of a particular transporter, no matter how indirect the phenotype. While there are many possible kinds of this assay, the simplest measures viability of human cells engineered for a dependency on a particular SLC. The best approach to circumvent redundancy and establish a robust genotype-phenotype relationship is to conduct a genetic screen in a cell line with inactivation of the targeted SLC. The simplest readout is growth/survival, hijacking the principle of synthetic lethality: while cell viability upon inactivation of either transporter A or transporter B is minimally affected, inactivation of both transporters leads to severly reduced fitness (Girardi et al., 2020b; Huang et al., 2020). In this setting, the fitness of the cell line with inactivated transporter B becomes dependent on transporter A, which provides a platform for screening for chemical modulators of the function of transporter A (Figure 11).

##### Technical Requirements and Level of Throughput

The assay requires the ability to genetically engineer a cell line of choice and the necessary tools are vectors allowing to express Cas9 and transporter-specific guides or chemical probes for the SLCs under investigation. If there is no prior knowledge on synthetic lethality to other SLCs, a prior genetic screen may be required. A cell-based viability assay is amenable to HTP screening.

##### Experimental Setup

Key requirement is establishing the unambiguous SLC-phenotype relationship. This includes the ability to engineer human cell lines to depend on a particular SLC gene and at least a second control cell line that is isogenic.

The first round of chemical screening is started with the engineered cell line dependent on the SLC of interest. All compounds which exhibit toxicity in the first round are screened in the secondary chemical screen, which includes additional isogenic control cell lines (e.g. WT parental cell line, and cell line dependent on the reciprocal SLC). Afterwards, all compounds which reduce viability only in the cell line dependent on the targeted SLCs are considered as a hit. Since this screening campaign enriches compounds targeting all genes with a synthetic lethal relationship to the targeted SLC, hits should be followed up for example with a binding assay to further filter compounds which interact physically with the targeted SLC.

##### Suitable Solute Carrier Families

The great advantage of this approach is that it is amenable to all SLC families, irrespective of their subcellular localization, topology or abundance, provided there is evidence of a genetic interaction between the SLC of interest and another protein and both are expressed in the cell line of choice. For example, strong negative genetic interactions were confirmed between SLC16A1 (MCT1) and SLC16A3 (MCT4), or between SLC25A28 (Mitoferrin-2) and SLC25A37 (Mitoferrin-1) (Girardi et al., 2020b). Using the parental cell line and the SLC16A1 KO as controls, differential activity of compounds against the SLC16A1 KO cell line yielded *bona fide* SLC16A3 inhibitors (Dvorak and Superti-Furga, unpublished).

##### Assay Advantages, Limitations and Approximate Costs

The main advantage of this assay is the straightforward readout based on cell viability, which is suitable for HTP screening. Since many of the genetic interactions are of reciprocal character, it is possible to counter screen for specificity by screening hits from the primary screen in reciprocal knock-out cells and WT cells. This approach should filter all compounds which affect viability of cells independently of the targeted SLC, and thus provide a fast path to compound specificity. The main limitation is the requirement of a strong genotype – phenotype association, which may require a prior dedicated genetic screen. Phenotypic assays can be applied also for screening for transporters of cytotoxic compounds (Girardi et al., 2020a).

## Discussion

The experimental methods and approaches that can be used to interrogate the function of SLC transporters are as diverse as this class of integral membrane proteins, with different phylogenetic origin, fold and transport mechanism. To enable drug discovery efforts, an extensive toolbox of technologies is required to study the diverse transport mechanisms utilized by SLC transporters as well as the broad nature of substrates transported.

**FIGURE 12:**
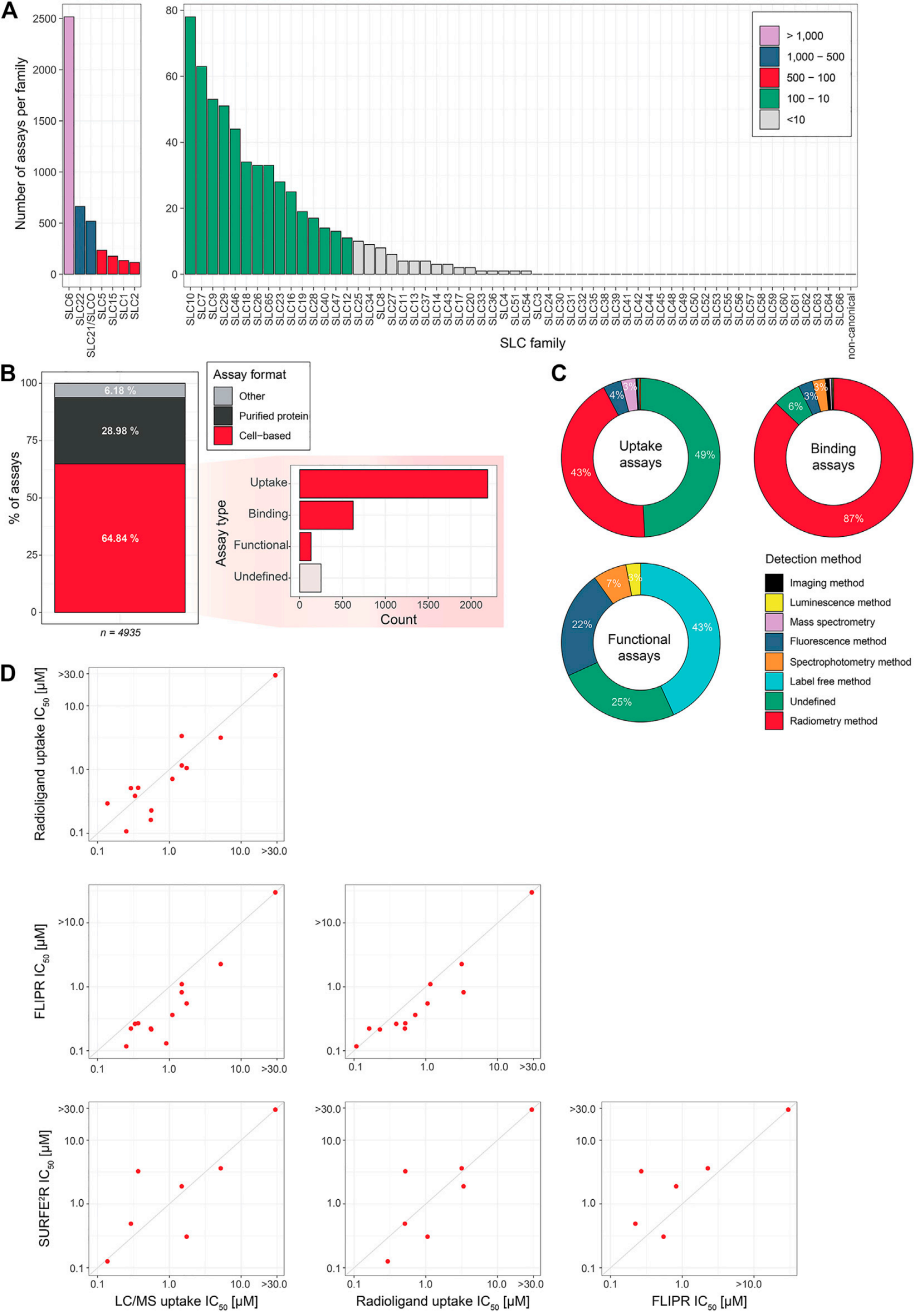
Overview and comparison of assay techniques in use. **(A)** Number of assays reported in ChEMBL per SLC family. **(B)** Distribution of assays based on assay format and assay type (cell-based assays only). Assay format was determined from the BAO label reported in the ChEMBL database (e.g. cell-based format and single protein format). Assay type was assigned according to manually created rules (see supplementary material) **(C)** Detection methods employed by each assay type (cell-based assays only). The detection method was assigned to the different categories based on manually created rules (see Supplementary table 2) **(D)** Comparison of IC_50_ values of SLC13A5 inhibitors obtained by different assays (Data retrieved from Huard et al. (2016); Pajor et al. (2016)); IC_50_ of >10, or >30 µM respectively, refers to the detection limit).

For an overview on the different assay techniques in use, we analyzed the assays for SLCs available in the ChEMBL database Version 28 (Davies et al., 2015; Gaulton et al., 2017) using KNIME (Berthold et al., 2009). In total, 4,935 assays (where each publication counts as one assay) were reported for 120 different SLCs (see Supplementary Table S2). These data are highly asymmetric, with the vast majority of assays performed only on a handful of SLC families, while there are less than 10 assays reported for more than a half of SLC families (Figure 12A). While this can be to some extent biased from the scope of the database, these observations are in line with a previously reported publication asymmetry in the SLC superfamily, in which a small fraction of SLCs are the object of a large proportion of literature on SLCs (César-Razquin et al., 2015). Our overview also showed that the majority of assays in ChEMBL were based on cell-based formats (Figure 12B). This is in line with the subject of the current review and reflects the challenges connected to purification and handling of complex membrane proteins.

We further characterized the assays according to the text of the assay description by ChEMBL. Text based rules were created manually to distinguish different assay types (e.g. uptake or binding) and detection methods (e.g. radiometry or label free methods). The full list of rules as well as the list of ChEMBL assays with assigned labels are given in the Supplementary Material. As cell-based assays are the focus of this review we concentrated on these assays for the overview given in Figures 12B,C. The majority of the cell-based assays are uptake assays, accounting for nearly 70%. When investigating the detection methods, we see that radiometry is heavily used both for uptake and binding assays. For the uptake assays we could not assign any detection method to almost 50% according to the assay description ("undefined"), but one can assume that radiometry was the method of choice in a large proportion of these cases as well. Only in the functional assays we do not see measurements of radioactivity. Instead, label free and fluorescence detection methods are used for measuring the outcome in functional cell-based assays. The total number of these assays is however still small, as functional assays make up only four percent of all cell-based assays (Figure 12C). We expect the frequency of functional assays to increase in the future due to more knowledge on less-studied SLCs, which should open the possibilities to study these SLCs particularly using functional assays. A better understanding of the SLC superfamily and its individual members will also facilitate the decision on which SLCs are amenable to which assay technologies.

The choice of an appropriate assay format is expected to change as a SLC drug discovery program progresses from its initial efforts to identify chemical matter that modulates the SLC transporter in the HTP screening phase where tens of thousands of compounds are screened, to file mining and virtual screening, and finally to SAR and lead development support. Multiple assay formats are often employed throughout the life of a program not only to address practical issues such as throughput and cost concerns but also to consider assay specific liabilities such as compound interference, cytotoxicity, off target interferences etc. Like with other target classes, as a hit matures to lead and preclinical and then clinical candidate, it will transit through several of these assays in different combinations.

To address the question if pharmacology of small molecules is influenced by choice of assay technology, we inspected previously obtained data on the potency of several SLC13A5 inhibitor chemotypes across different technologies in a cellular system in which SLC13A5 was overexpressed (Huard et al., 2016; Pajor et al., 2016): uptake of radiolabeled citrate, uptake of citrate monitored by LC/MS, uptake of co-transported sodium using FLIPR to monitor membrane potential and solid supported based electrophysiology using SURFE^2^R (Figure 12D). In sum, the potency of the SLC13A5 inhibitors correlated across all the technologies tested. This suggests that data obtained with one assay should represent a reliable starting base for another assay. It also suggests that modern assay technology can be calibrated in such a way to be robust. While we consider this observation rewarding and promising, this does not necessarily imply that the discovery efficiency should be equal, as sensitivity may differ, and certain molecules may have properties that manifest differentially in the different assays. All in all, based on the variety and robustness of available assays, SLCs should be considered an attractive and amenable target class.

## Future Perspectives

From our perspective the future of SLCs as major target class in drug discovery seems certain. The number of reagents, tools, assays and publications are increasing constantly. Only in the last two years we counted three new biotech start-ups focused entirely on SLCs, the first of what surely is likely to be a whole generation of new businesses. The majority of pharmaceutical companies either already include SLC targets in their project portfolio or are rapidly evaluating the opportunity. Among the novel drugs with new mechanism of action reported for 2020, SLCs feature prominently (Avram et al., 2021). Though the dataset is small, it heralds the beginning of a new phase in SLC pharmacology. The RESOLUTE consortium intends to empower the scientific community with reagents, datasets and assays and invites it to contribute to exploit transporters more broadly for drug discovery. Through their action, transporters link metabolism to a variety of cellular and organismal processes, all relevant to disease. At a yet more futuristic and ecological level, transporters of roughly the same kind, across the animal, plant, fungal and microbial kingdom, ensure flow and integration of chemical matter in a global way. Thus, what we are learning about the assays required in the field of SLC transporter pharmacology for curing human diseases, may turn out to be useful also for nutrition, animal and crop production, managing of microbial communities and more.
